# Completion of the Total Synthesis of Several Bioactive Sarpagine/Macroline Alkaloids including the Important NF-κB Inhibitor *N*_4_-Methyltalpinine

**DOI:** 10.3390/molecules27051738

**Published:** 2022-03-07

**Authors:** Md Toufiqur Rahman, Veera Venkata Naga Phani Babu Tiruveedhula, Michael Rajesh Stephen, Sundari K. Rallapalli, Kamal P. Pandey, James M. Cook

**Affiliations:** Department of Chemistry and Biochemistry, University of Wisconsin Milwaukee, Milwaukee, WI 53211, USA; mdrahman@uwm.edu (M.T.R.); tiruvee2@uwm.edu (V.V.N.P.B.T.); smichaelrajesh@gmail.com (M.R.S.); rallapallisk@gmail.com (S.K.R.); kppandey@uwm.edu (K.P.P.)

**Keywords:** sarpagine alkaloids, macroline alkaloids, macrocarpine F, macrocarpine G, talpinine, *O*-acetyltalpinine, N(4)-methyltalpinine, NF-κB inhibitor, anticancer alkaloids, N-dealkylation, bioactive alkaloids

## Abstract

The unification of the general synthetic strategy regarding the important and emerging group of C-19 methyl-substituted sarpagine/macroline alkaloids has culminated in the completion of the total synthesis of several bioactive alkaloids. Key transformations include an ACE-Cl mediated late-stage N(4)-demethylation and an anhydrous acid-mediated intramolecular quaternary hemiaminal formation between a tertiary amine and an aldehyde function to allow efficient access to several biologically important alkaloids from this group. Herein, the enantiospecific total synthesis of the first known sarpagine/macroline alkaloid with NF-κB inhibitory activity, N(4)-methyltalpinine (as a chloride salt), as well as the anticancer alkaloids talpinine, *O*-acetyltalpinine, and macrocarpines F–G, are described.

## 1. Introduction

The sarpagine/macroline/ajmaline family represents an important class of biosynthetically related monoterpene indole alkaloids [1,2,3]. Sarpagine, ajmaline, and macroline individually are eponymous with respect to their corresponding subclass of alkaloids. To date, this family comprises more than 300 monomeric and about 100 bisindole alkaloids [2,4,5,6]. A number of alkaloids from this family have been reported to possess important biological properties, such as anticancer, antileishmanial, antiarrhythmic, and antimalarial activities, which is not surprising since these alkaloids occur primarily in various plant species of the *Apocynaceae* family that have been used in traditional or folk medicines for centuries [4,7,8,9,10,11].

The C-19 methyl-substituted sarpagine/ajmaline sub-family of alkaloids is a growing ensemble of alkaloids with a common structural feature, i.e., alkaloids having a stereogenic methyl group at the C-19 position of their biogenetic architecture. To date, this group has been populated with more than seventy alkaloids [8,12]. Despite this common structural feature, notable diversity in substitution patterns, and oxidation states, very few syntheses have been reported for this sub-group [13,14,15]. In addition, as usual, the paucity of isolated material has impeded the study of medicinal properties.

**Figure 1:**
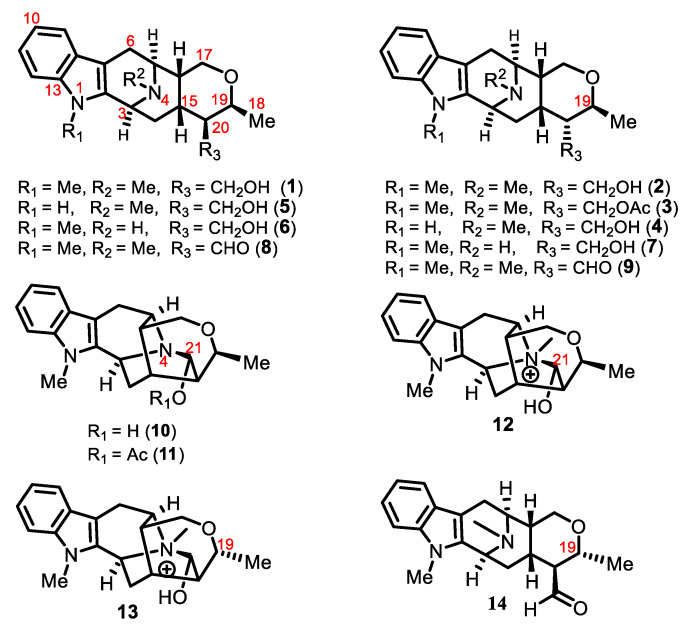
Representative examples of C-19 methyl-substituted sarpagine/macroline alkaloids.

A general and unified synthetic approach regarding this emerging group has been of interest due to the important biological activities as well as the interesting structures of a number of alkaloids from the *Apocynaceae* [8]. One of the important outcomes of the general strategy developed in Milwaukee is the improved and readier access to the core sarpagine/ajmaline architecture via an ambidextrous Pictet–Spengler/Dieckmann protocol, which enabled access to either enantiomer starting from either of the tryptophan chiral auxiliaries, providing access to the natural alkaloids from the cheaper and DNA-encoded L-tryptophan or D-tryptophan [12,16,17,18]. The proof of concept has been illustrated by accessing the key intermediates for both the natural and unnatural enantiomers of the natural alkaloids in an enantiospecific manner [16,17,18]. In addition, to date, the strategy has been utilized in the total synthesis of various alkaloids from this family with various ring systems, substitution patterns, and oxidation states across the familial skeleton of these alkaloids [13,14,15,16,17,18,19]. Some representative alkaloids from this sub-group are illustrated in Figure 1. Macrocarpines A–G (**1**–**7**) are a class of closely related macroline-type alkaloids. Talcarpine (**8**), *N*_4_-methyl-*N*_4_,21-secotalpinine (**9**), and their corresponding *N*_a_-H versions served as the precursors for alkaloids **1–7** [15,19]. The total synthesis of macrocarpines A–E (**1**–**5**) has been reported previously, along with the precursors (Figure 1) [15,19]. Macrocarpines F (**6**) [20] and G (**7**) [20] are *N*_b_-demethylated versions of macrocarpines A (**1**) and B (**2**), respectively [21]. Talpinine (**10**), a macroline-derived sarpagine alkaloid has been known for years as a key base [20,22]. In talpinine (**10**) and closely related alkaloids, the C-20 of the macroline scaffold is directly connected to the *N*_b_-nitrogen atom providing the linkage characteristic to the sarpagines which is absent in the common macroline-type alkaloids [1,2]. Reported in the recent literature are several talpinine-related alkaloids and their biological activities. Among them, talpinine (**10**) and *O*-acetyltalpinine (**11**) exhibited cytotoxicity (IC_50_ = 14–22 µg/mL) in reversing multidrug resistance in drug-resistant KB/VJ300 cells, [20] while *N*_4_-methyltalpinine (**12**) showed promising NF-κB (p65) inhibitory activity (ED_50_ = 1.2 µM). *N*_4_-Methyltalpinine (**12**) is a quaternary ammonium C-19 methyl-substituted sarpagine-related alkaloid and is the first sarpagine-type indole alkaloid to possess NF-κB inhibitory activity [23]. The counter anion was not reported during the isolation of **12** [23]. It is believed that in the native form of **12** the counter anion is probably a mixture of what could be naturally occurring acids. During the isolation of such quaternary alkaloids, it is possible to fix the counter anion (e.g., Cl^−^) by passage through an appropriate ion-exchange column [24]. In this regard, during the synthesis of **12**, the counter anion would arise from the respective alkyl halide that would be used in the quaternization step. Talcarpine (**8**) and its C-20 antipode **9** are known for their antimalarial [25] and antileishmanial [23] properties, respectively. While most of the alkaloids in the C-19 methyl-substituted sarpagine/macroline alkaloids bear an (*S*) configuration at the C-19 carbon atom, a few alkaloids with the (*R*) configuration at C-19, namely, *N*(4)-methyl-19-*epi*-talpinine (**13**), [20] 19-*epi*-talcarpine (**14**), [20] and deoxyperaksine [26] (not shown here), are also known (Figure 1). None of the C-19(*R*) alkaloids have any known biological activity, to date, while their C-19(*S*) counterparts (e.g., **8** and **12**) possess important bioactivities [23,25]. This implies that the C-19(*S*) configuration might be a key structural motif for the bioactivity of these alkaloids. As a result, from a medicinal chemistry point of view, alkaloids with the C-19(*S*) are logically more promising than their C-19(*R*) antipodes. Accordingly, the development of a practical synthetic strategy for the C-19(*S*) alkaloids has gained more attention [8,12]. Herein, we report the first enantiospecific total synthesis of the potent NF-κb inhibitor and quaternary ammonium containing alkaloid *N*_4_-methyltalpinine (**12**) as a chloride salt, along with the anticancer alkaloids talpinine (**10**) and *O*-acetyltalpinine (**11**), as well as macrocarpines F and G (**6–7**).

## 2. Results and Discussion

**Scheme 1:**
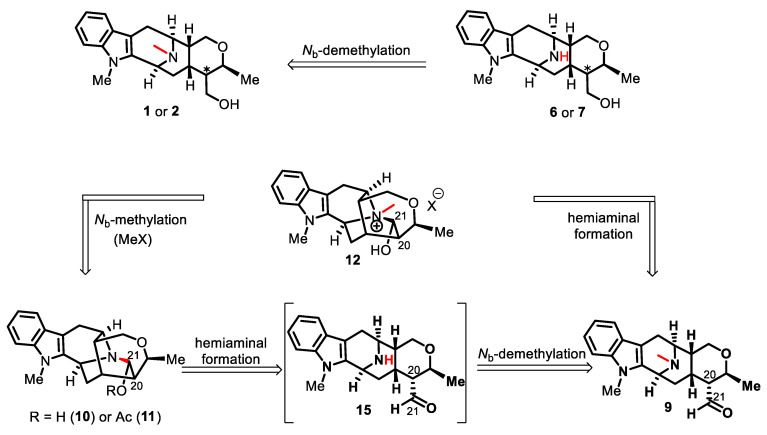
Retrosynthetic strategy for the synthesis of C-19 methyl-substituted alkaloids.

Since the *N*_a_-CH_3_, *N*_b_-CH_3_ alkaloids, and macrocarpines A–B (1–2) were in hand from previous work, [15] it was felt the *N*_b_-H substitution in macrocaprines F (**6**) and G (**7**) would be accessible via an *N*_b_-demethylation process from **1** [21] and **2** [21], respectively (Scheme 1). Talpinine (**10**) [22] is a macroline-derived sarpagine-type alkaloid containing a hemiaminal (carbinolamine) function in which the N(4) atom of the sarpagine architecture is directly connected to the C(21) atom of the macroline system. This provides an additional ring, as well as increasing the rigidity of the system due to the quinuclidine moiety. Retrosynthetically, the hemiaminal function at C-21 would originate from an intramolecular cyclization between the secondary *N*_b_-nitrogen atom (as in **15**) with the C-20α formyl function (Scheme 1). The secondary amine **15** would be available from the tertiary amine **9** via dealkylation. The same *N*_b_-demethylation reaction employed for the synthesis of **6** and **7** should also be useful in this case. On the other hand, for the important quaternary ammonium alkaloid **12** containing a methyl function at the *N*_b_-nitrogen atom of talpinine (**10**), the most obvious precursor would be **10** itself, which would be accessible by means of an *N*_b_-methylation process with a methyl halide (Scheme 1). Although the counter anion for the quaternary ammonium ion in the natural sample was not known, [23] the synthetic *N*_4_-methyltalpinine would have the counter anion corresponding to the methyl halide used. Another possible way of accessing *N*_4_-methyltalpinine would be the intramolecular reaction between the tertiary *N*_b_-amine nitrogen atom with the C-21 carbonyl function of *N*_4_-methyl-*N*_4_,21-secotalpinine **9**. However, potential steric hindrance and conformational restriction of the tertiary *N*_b_-nitrogen atom could deter it from reacting with the aldehyde function to furnish the hemiaminal (carbinolamine) function present in the desired *N*_4_-methyltalpinine. The carbonyl function in **9** could be further activated towards nucleophilic addition by means of a Lewis or Brønsted acid catalyst.

As planned, the focus was initially on the search for and optimization of a facile and robust method for the *N*_b_-dealkylation process. Late-stage transformation towards important alkaloids sometimes occurs with limited amounts of the required precursors in hand (which albeit are alkaloids themselves), therefore, as mentioned, the *N*_b_-demethylation process must be robust. The *N*_b_-methyl function is inert to many chemical transformations and aggressive reagents. Consequently, it is considered to be a persistent protecting group for amines [27,28]. In addition, it is an additional challenge to remove a methyl group on amines in functionally rich and sterically hindered systems, such as **1** and **2**. In order to remove the *N*_b_-methyl group regioselectively, it was decided to attempt a number of available methods present in the literature. The use of cyanogen bromide (Von Braun reaction) [29] and carbonochloridates [30] (chloroformates) are well-known for regioselective dealkylation of alkylamines. It was also reported that the chloroformates are one of the best methods for this purpose due to better selectivity, as well as cleaner and milder reaction conditions. In addition, one looked for inspiration from similar transformations in related systems. Inspired by the methods in the total synthesis of the oxindole alkaloid isoalstonisine by Fonseca [31], fortunately, the chloroformate was successfully used to *N*-dealkylate the *N*_b_-methyl group in a congested system at the final stage of the synthesis. It was then decided to employ the well-known *N*-dealkylation process developed by Olofson et al. [32]. This process employs an excess of ACE-Cl in 1,2-DCE at reflux. The so formed quaternary ammonium carbamate, upon refluxing in methanol, followed by a basic work-up, would provide the desired *N*_b_-demethylated products, i.e., **6** and **7**.

**Scheme 2:**
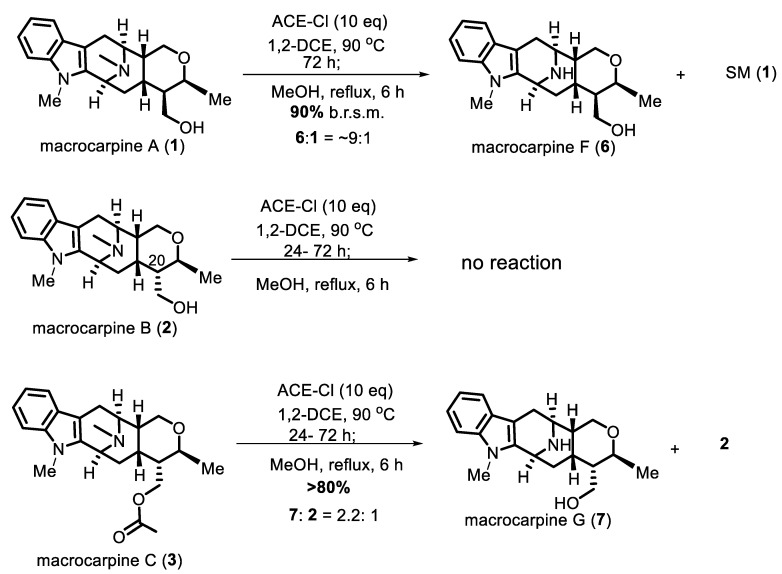
Completion of the total synthesis of **6** and **7** via the late-stage demethylation process.

As planned, macrocarpine A (**1**) and B (**2**) (individually) were reacted with **10** equivalents of ACE-Cl in refluxing 1,2-dichloroethane. After that, the reaction mixtures were dissolved in dry methanol and heated at reflux, and this was followed by a basic work-up with cold aq **1** *N* NaOH (Scheme 2). The C-20*β* hydroxymethyl compound, macrocarpine A (**1**), furnished the *N*_b_-demethylated secondary amine, macrocarpine F (**6**, LRMS: [M + H]^+^ = 327), along with unreacted starting material **1**, which resulted in a 90% yield (based on recovered starting material). An LC–MS analysis of the reaction mixture indicated that the desired product **6** and the starting material **1** were present in a ratio of 88:12. On the other hand, it was observed that the C-20*α* hydroxymethyl compound, macrocarpine B (**2**), remained unchanged (LRMS: [M + H]^+^ = 341) even after prolonged heating at reflux (up to 72 h), i.e., the *N*_b_-methyl base **2** did not form the quaternary ammonium carbamate salt, hence the subsequent decarboxylation did not proceed. Consequently, the *O*-acetyl variant of 2, macrocarpine C (3, MW = 382.5), was subjected to the same conditions used for 2. It was observed that the desired *N*_b_-demethylated secondary amine, macrocarpine G (**7**), formed (LRMS: [M + H]^+^ = 327), and this was accompanied by the deacetylated compound **2** (LRMS: [M + H]^+^ = 341), providing an overall yield of >80%.

**Figure 2:**
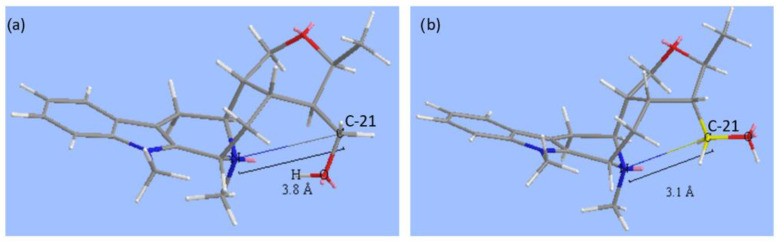
Energy-minimized (MM2) models using Chem3D Pro 12.0 of (**a**) macrocarpine A (**1**) and (**b**) macrocarpine B (**2**). The C-21 atom in macrocarpine B is generating greater steric congestion to the oncoming ACE-Cl electrophile toward the N(4) nitrogen atom. In addition, the C-21 in **2** is closer than the C-21 in **1** to the N(4) nitrogen atom, which suggests stronger intra-molecular H-bonding between the C-21 (OH) with the N(4) nitrogen atom in **2**.

From these observations, it was concluded that the C-20α hydroxymethyl group in tertiary amine **2** was too close to the amine function, which created some steric congestion and probably hindered the amine function from reacting with the chloroformate. More importantly, from molecular modeling (see Figure 2), it was clear that a hydrogen bond between the tertiary amine function in **2** with the primary alcohol would also retard the amine function from reacting with the chloroformate. This was evident from the fact that both macrocarpine A **1** and the *O*-acetyl version of macrocarpine B (macrocarpine C, 3) did react to form the desired *N*_b_-demethylated products **6** and **7**, respectively. The spectroscopic properties of **6** and **7** were in excellent agreement with the natural alkaloids [20] (see Section 3).

**Scheme 3:**
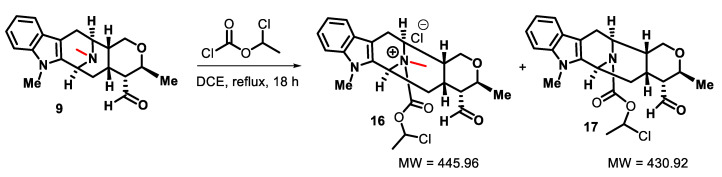
*N*_b_-demethylation of **9** using ACE-Cl.

**Scheme 4:**
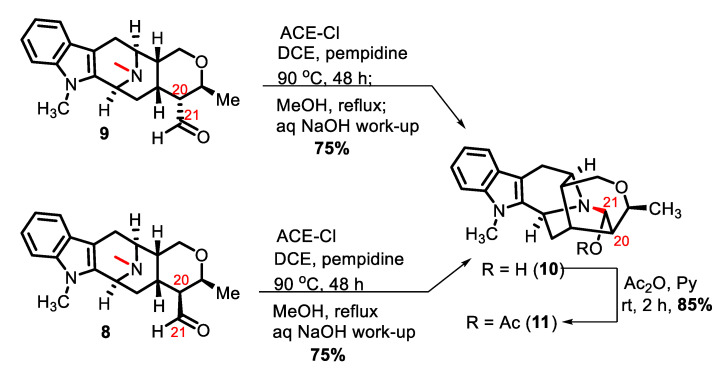
Synthesis of talpinine (**10**) and *O*-acetyltalpinine (**11**).

Since the desired demethylation was executed effectively, the C-20α aldehyde **9** was also subjected to the Olofson [32] *N*_b_-demethylation conditions, which furnished the demethylated secondary amine **15** in situ, and it underwent cyclization, subsequently, in an intramolecular fashion, to form the desired hemiaminal present in talpinine **10**. During the initial trials it was observed that the starting tertiary amine **9** was somewhat unreactive with the chloroformate (ACE-Cl) and the conversion was very slow. It was felt that using a bulky and non-nucleophilic base, such as pempidine (1,2,2,6,6-pentamethylpiperidine) [33], would facilitate the carbamate formation at the initial stage of the demethylation process by scavenging any residual protons present in the reaction solution. Using a stoichiometric amount of pempidine and an excess of ACE-Cl in DCE at reflux (for 18 h), it was observed that the corresponding carbamates (**16**, LRMS: M^+^ = 445; **17**, LRMS: [M + H]^+^ = 431) formed, but the starting tertiary amine (**9**, LRMS: [M + H]^+^ = 339) still remained (Scheme 3). This indicated that the first intermediate, **16**, which was formed by the reaction between the tertiary amine nitrogen atom with the ACE-Cl carbonyl function, was present as the major product in the reaction mixture (after 18 h), while the *N*_b_-demethylated carbamate **17** was present as the minor product. Gratifyingly, this observation indicated that the reaction was progressing, albeit slowly. Accordingly, the reaction mixture was subjected to prolonged heat (up to 42 h), which completed the conversion, as indicated by the absence of the starting material **9** upon analysis by LC–MS. After the subsequent decarboxylation reaction in refluxing methanol was followed by an alkaline work-up, this process furnished the secondary amine (see **15** in Scheme 1). The amine *N*_b_-nitrogen atom reacted with the C-21 formyl function, as above, ultimately to form the hemiaminal present in talpinine **10** (Scheme 4) in 75% yield. Examination of the ^1^H NMR spectrum confirmed the absence of the formyl function (at *δ* 9.44 ppm) and the presence of the H-21 proton (at carbinolamine carbon, C-21) at *δ* 4.71 ppm.

The C-20β aldehyde function containing indole base **8** was also subjected to the same conditions to check whether the stereochemistry at the C-20 function played a significant role in the rate of initial carbamate formation. It was found that talcarpine **8** also underwent demethylation and furnished the same product, talpinine **10** in 75% yield. This indicated that the C-20 aldehyde undergoes epimerization under these conditions to the *α*-stereochemistry and then cyclizes (Scheme 4). Simple acetylation of talpinine **10** using standard methods furnished *O*-acetyltalpinine **11** in 85% yield. The spectral and optical properties of synthetic **10** and **11** were in excellent agreement with the values reported in the literature [22,34] for the corresponding natural products.

**Scheme 5:**
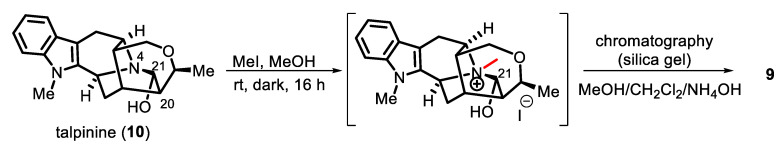
Attempted synthesis of *N*_4_-methyltalpinine **12** from talpinine **10**.

**Figure 3:**
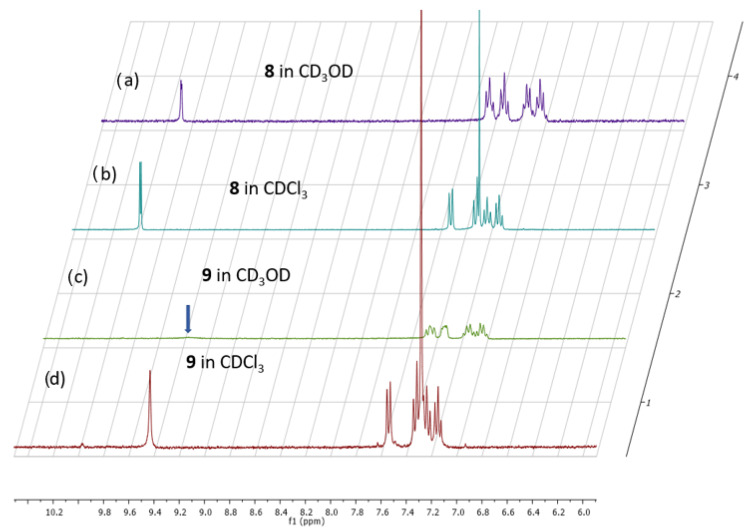
Comparison of the ^1^H NMR (300 MHz) spectra of talcarpine **8** and *N*_4_-methyl-*N*_4_,21-secotalpinine **9** in CD_3_OD and CDCl_3_ (δ 10.5 to 6.0 ppm). ^1^H NMR spectrum of (**a**) **8** in CD_3_OD; (**b**) **8** in CDCl_3_; (**c**) **9** in CD_3_OD; (**d**) **9** in CDCl_3_. The arrow indicates the position of the broadened aldehyde signal of **9** in CD_3_OD.

At this point, as planned, talpinine **10** was treated with iodomethane in methanol at room temperature in the dark for 16 h (Scheme 5). A ^1^H NMR spectrum of the reaction mixture in deuterated methanol indicated the disappearance of the aldehydic proton (at δ 9.44 ppm), whereas a broad multiplet at *δ* 5.0–4.9 ppm appeared, which was expected for the hemiaminal proton (H-21 on the carbinolamine carbon, C-21). This result was encouraging; however, the NMR spectrum of the crude reaction mixture was not clean enough for full characterization and for comparison with the natural alkaloid [23]. Consequently, chromatography (silica gel, CH_2_Cl_2_/MeOH/28%NH_4_OH (aq.); 94:5:1) was attempted in order to obtain a pure sample of synthetic *N*_4_-methyltalpinine (**12**). Unfortunately, the compound that was isolated by chromatography lacked the hemiaminal proton peak at ~δ 5 ppm as well as the aldehydic proton peak at δ 9.44 ppm. Intrigued by this result, attempts were made to identify this product. The product was found to be identical to *N*_4_-methyl-*N*_4_,21-secotalpinine **9** in deuterated methanol. One important observation was made. In deuterated methanol the aldehyde peak of **9** (Figure 3c) was not well defined in comparison to the ^1^H NMR spectrum of **8** in CD_3_OD (Figure 2a). On the other hand, in CDCl_3_ the aldehyde peak of both **8** (Figure 3b) and **9** (Figure 3d) was well-defined.

This result indicated that while the C-20*β* aldehyde in talcarpine **8** remained as an aldehyde moiety in methanol, there was an equilibrium mixture in the case of *N*_4_-methyl-*N*_4_,21-secotalpinine **9** (Figure 3). Furthermore, it was observed that the aldehyde peak broadened or sharpened in a temperature-dependent manner. At higher temperatures, the aldehyde peak was found to be broader than the corresponding aldehyde peak at lower temperatures (not shown here).

**Scheme 6:**
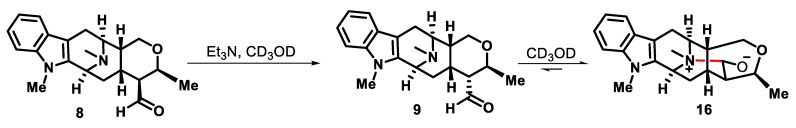
Epimerization of talcarpine **8** into *N*_4_-methyl-*N*_4_,21-secotalpinine **9** under basic conditions.

Furthermore, it was determined that the aldehyde peak broadened after the epimerization of talcarpine **8** into **9**. Talcarpine **8** was treated with triethylamine in methanol at room temperature. It was observed that the aldehyde peak of **8** (at δ 9.9 ppm) gradually diminished and a broad peak corresponding to the C-20*α* aldehyde proton appeared at δ ~9.3 ppm. This experiment indicated that the *β*-aldehyde function in talcarpine **8** epimerized in the presence of a base and gradually formed the corresponding α-aldehyde **9**, which is the thermodynamically more stable epimer. As soon as the α-aldehyde **9** was formed, it interacted with the tertiary amine, which was in the vicinity and formed an equilibrium favoring the cyclized form. As a result, this altered the sharp peak for the aldehyde to a broader peak (Scheme 6).

**Scheme 7:**
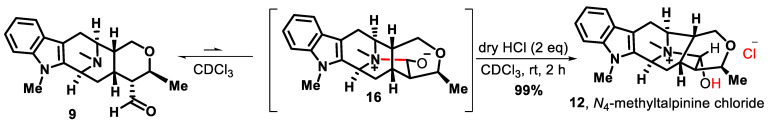
Formation of *N*_4_-methyltalpinine (**12**) from **9** via **16**.

**Figure 4:**
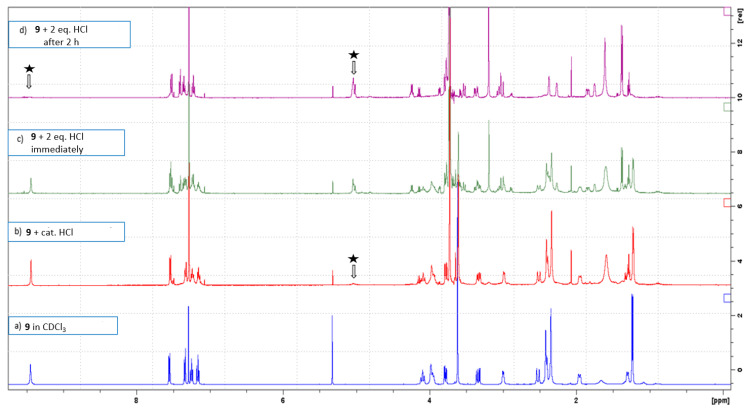
Progress of the reaction of dry HCl with indole base **9** (^1^H NMR, CDCl_3_, 300 MHz) to form *N*_4_-methyltalpinine (**12**). ^1^H NMR of (**a**) **9**, (**b**) **9** + HCl (cat.), (**c**) **9** + HCl (2 eq) immediately after addition, and (**d**) **9** + HCl (2 eq) after 2 h. The star symbols mark the positions of changes in important signals.

Given the experiments described above, it was felt that the indole base **9** would stay principally in a Zwitterionic form with **16** in methanol (Scheme 6), while in chloroform the cyclized form **16** would be present in a small amount while the equilibrium favored mostly the open form **9** (Scheme 7). In the presence of dry HCl in solution, the oxygen atom would be protonated and the chloride ion would act as the counter anion for the quaternary ammonium nitrogen atom, shifting the equilibrium towards the closed form **16** (i.e., **12**). This would lead to an irreversible formation of the desired stable *N*_4_-methyltalpinine **12** as a chloride salt (Scheme 7) if silica gel chromatography was avoided. Consequently, the base **9** was stirred with anhydrous HCl (4.0 M solution in dioxane) at room temperature. Then deuterated chloroform was used as the solvent instead of CD_3_OD to avoid any peak overlap with the peak at δ 4.87 ppm (residual moisture) with the desired H-21 peaks at δ 5.00–4.95 ppm. After adding a catalytic amount of dry HCl, a small broad peak at δ 5.0 ppm appeared, which indicated that the conversion had begun, albeit in very small amounts (Figure 4b). After adding 2 equivalents of anhydrous HCl it was observed that the multiplet at δ 5 ppm increased in intensity, while the aldehydic peak at δ 9.44 ppm began to diminish in intensity (Figure 4c). After standing at room temperature for an additional 2 h, examination of the ^1^H NMR spectrum indicated that the aldehydic peak was completely gone and the spectrum appeared much cleaner (Figure 4d). After that, the solvent was removed under reduced pressure and the alkaloid was dissolved in deuterated methanol for comparison with the literature values (see Section 3). A ^1^H NMR spectrum of the compound in CD_3_OD was found to be identical to one found in the literature [23]. All other spectroscopic and optical rotation values were in excellent agreement with the literature values natural [23] for the desired alkaloid *N*_4_-methyltalpinine **12**. (Caution: in Zwitterionic molecules, such as **12**, it is best to avoid silica gel chromatography).

## 3. Experimental

### 3.1. General Experimental Considerations

All reactions were carried out under an argon atmosphere with dry solvents using anhydrous conditions unless it is stated otherwise. The solvents (THF, DMF, toluene, DCM, MeCN, and MeOH) were dried using an Innovative Technology solvent purification system, Pure Solv^TM^. Occasionally, tetrahydrofuran was freshly distilled from Na/benzophenone ketyl prior to use. Dichloromethane was distilled from calcium hydride prior to use. Methanol was distilled over magnesium sulfate. Benzene was distilled over CaH_2_. Reagents were purchased of the highest commercial quality and used without further purification unless otherwise stated. Thin layer chromatography (TLC) was performed on UV active silica gel plates, 200 μm, aluminum-backed and UV active alumina N plates, 200 μm, F-254 aluminum-backed plates. Flash and gravity chromatography were performed using silica gel P60A, 40–63 μm, basic alumina (Act I, 50–200 μm), and neutral alumina (Brockman I, ~150 mesh). TLC plates were visualized by exposure to short wavelength UV light (254 nm). Indoles were visualized with a saturated solution of ceric ammonium nitrate (CAN) in 50% phosphoric acid. The ^1^H NMR data are reported as follows: chemical shift, multiplicity (br s = broad singlet, s = singlet, d = doublet, t = triplet, q = quartet, quin = quintet, dd = doublet of doublets, dt = doublet of triplets, ddd = doublet of doublet of doublets, td = triplet of doublets, qd = quartet of doublets, m = multiplet), integration, and coupling constants (Hz). The ^13^C NMR data are reported in parts per million (ppm) on the δ scale. The low-resolution mass spectra (LRMS) were obtained as electron impact (EI, 70eV) and as chemical ionization (CI) using a magnetic sector (EBE) analyzer. HRMS was performed with electrospray ionization (ESI) using a TOF analyzer, electron impact (EI) using a trisector analyzer and atmospheric pressure chemical ionization (APCI) using a TOF analyzer. Optical rotations were measured on a JASCO Model DIP-370 polarimeter.

### 3.2. Macrocarpine F (***6***)

The indole **1** (3 mg, 0.009 mmol) was dissolved in dry 1,2-dichloroethane (2 mL) in a thick-walled vessel that could be sealed with a screw cap. The ACE-Cl (1-chloroethyl chloroformate, 12.6 mg, 0.09 mmol) was added to the above solution at 0 °C under argon. The reaction vessel was sealed and heated at 90 °C (oil bath) for 72 h. The reaction was then cooled to room temperature and the solvent was removed under reduced pressure. Then, distilled methanol (5 mL) was added to the residue and the solution that resulted was heated at reflux under argon for 6 h with stirring. After that, the solvent was removed under reduced pressure and the residue was dissolved in EtOAc (5 mL) and brought to pH 8 with cold aq **1** *N* NaOH. The organic layer was separated and the aq layer was extracted with additional EtOAc (2 × 5 mL). The combined organic layers were washed with brine and dried (K_2_CO_3_). The solvent was removed under reduced pressure to give a brown residue. The residue was purified by column chromatography (silica gel, CH_2_Cl_2_/MeOH; 20:1) to yield macrocarpine F **6** as a colorless residue (2.3 mg, 80%). The spectroscopic data for the synthetic alkaloid were in excellent agreement with those for the natural product [20]. For ^1^H NMR results, see Table 1; for ^13^C NMR results: see Table 1. (Note: complete structural assignment was carried out based on ^1^H, ^13^C, DEPT-135, COSY, and HSQC NMRs; see Supplementary Materials for NMR spectra.) HRMS: (ESI) *m/z* [M + H]^+^ calculated for C_20_H_27_N_2_O_2_ 327.2067, found 327.2060; *R*_f_: 0.1 (silica gel, CH_2_Cl_2_/MeOH; 20:1). (Note: the optical rotation was not measured due to the loss of material during re-purification.)

### 3.3. Macrocarpine G (***7***)

**Figure 5:**
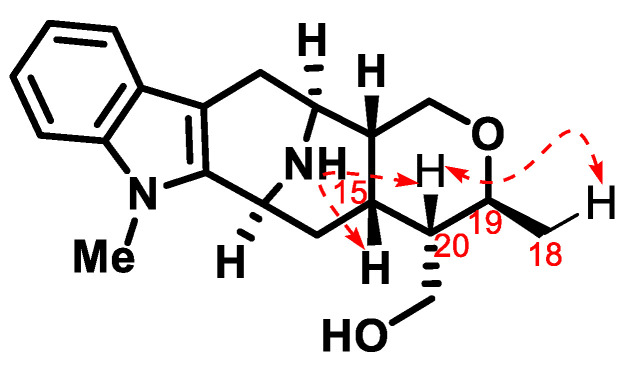
Selected NOE that confirms the C-20 stereochemistry of macrocarpine G (**7**).

The indole 3 (4 mg, 0.01 mmol) was dissolved in dry 1,2-dichloroethane (3 mL) in a thick-walled vessel that could be sealed with a screw cap. The ACE-Cl (1-chloroethyl chloroformate, 14.9 mg, 0.10 mmol) was added to the above solution at 0 °C under argon. The reaction vessel was sealed and heated at 90 °C (oil bath) for 72 h. The reaction mixture was then cooled to room temperature and the solvent was removed under reduced pressure. Then, distilled methanol (5 mL) was added to the residue and the solution that resulted was heated at reflux under argon for 6 h with stirring. After that, the solvent was removed under reduced pressure and the residue was dissolved in EtOAc (5 mL) and brought to pH 8 with cold aq **1** *N* NaOH. The organic layer was separated and the aq layer was extracted with additional EtOAc (2 × 5 mL). The combined organic layers were washed with brine and dried (K_2_CO_3_). The solvent was removed under reduced pressure to give a brown residue. The residue was purified by column chromatography (silica gel, CH_2_Cl_2_/MeOH; 20:1) to yield macrocarpine G (**7**) as a colorless residue (1.9 mg, 55%) accompanied by **2** (0.9 mg, 25%). The optical rotation and spectroscopic data were in agreement with those for the natural product [20]. For ^1^H NMR results: see Table 2; *R*_f_: 0.1 (silica gel, CH_2_Cl_2_/MeOH; 20:1); [α]_D_^25^: Synthetic = +12.0 (c 0.5, CHCl_3_); Natural [20]: = +7 (c 1.1, CHCl_3_); HRMS: (ESI) *m*/*z* [M + H]^+^ calculated for C_20_H_26_N_2_O_2_ 327.2067, found 327.2074. (Note: A ^13^C NMR measurement was attempted but due to the very small amount of sample available, it was not successful, even after a longer experiment time. The full structural assignment was carried out based on ^1^H, COSY, and NOESY NMR spectroscopy (see Figure 5 for important NOE confirmation) and comparison of the spectra with those of the natural alkaloid^1^.)

### 3.4. Talpinine ***10***

The indole **8** or **9** (6 mg, 0.018 mmol) was dissolved in dry 1,2-dichloroethane (4 mL) in a thick-walled vessel that could be sealed with a screw cap. The ACE-Cl (1-chloroethyl chloroformate, 25.3 mg, 0.18 mmol) and pempidine (2.7 mg, 0.018 mmol) were added to the above solution at 0 °C under argon. The reaction vessel was sealed and heated at 90 °C (oil bath) for 42 h. The reaction was then cooled to room temperature and the solvent was removed under reduced pressure. Then, distilled methanol (5 mL) was added to the residue and the solution that resulted was heated at reflux under argon for 6 h with stirring. After that, the solvent was removed under reduced pressure and the residue was dissolved in EtOAc (5 mL) and brought to pH 8 with cold aq **1** *N* NaOH. The organic layer was separated and the aq layer was extracted with additional EtOAc (2 × 5 mL). The combined organic layers were washed with brine and dried (K_2_CO_3_). The solvent was removed under reduced pressure to give a brown residue. The residue was purified by chromatography (silica gel, CH_2_Cl_2_/MeOH; 20:1) to yield talpinine **10** as a colorless oil (4.3 mg, 75%). The spectral data were in excellent agreement with those of the natural product [22,34] and the previous synthesis by Yu et al. For ^1^H NMR results: see Table 3; for ^13^C NMR: see Table 3; [α]_D_^25^ = −30.0 (c 0.2, CHCl_3_); Natural [22]: [α]_D_^25^ = −30 (c 0.302, CHCl_3_); HRMS: (ESI) *m/z* [M + H]^+^ calculated for C_20_H_25_N_2_O_2_ 325.1911, found 325.1919; *R*_f_: 0.1 (silica gel, CH_2_Cl_2_/MeOH; 20:1).

### 3.5. O-Acetyltalpinine (***11***)

To a mixture of Ac_2_O and pyridine (1:1, 0.5 mL), talpinine **10** (1 mg, 0.003 mmol) was added at room temperature under argon. The solution that resulted was stirred at room temperature for 2 h. After that, a cold solution of saturated aq Na_2_CO_3_ (2 mL) was added to the above reaction. The solution was extracted with CH_2_Cl_2_ (3 × 3 mL). The combined organic layers were washed with brine. The solvent was removed under reduced pressure and the residue was purified by column chromatography (silica gel) in a Pasteur pipette with 0–3% MeOH in CH_2_Cl_2_ to afford *O*-acetyltalpinine **11** (0.96 mg, 85%) as a colorless waxy solid. The spectral data for **11** were identical to those of the natural product [20]. For ^1^H NMR results: see Table 4; for ^13^C NMR: see Table 4; *R*_f_: 0.3 (silica gel, CH_2_Cl_2_/MeOH; 20:1); Synthetic: [α]_D_^25^ = −9.1 (c 0.12, CHCl_3_)*; Natural [20]: [α]_D_^25^ = −8 (c 0.2, CHCl_3_); HRMS: (ESI) *m/z* [M + H]^+^ calculated for C_22_H_27_N_2_O_3_ 367.2016, found 367.1995.

(Note: There was an unidentified minor impurity in the synthetic 11, as indicated by examination of the ^1^H NMR spectrum; the compound appeared as a single spot on TLC (silica gel). In addition, only the desired compound’s (**11**) mass (LRMS [M + H]^+^ = 367) was observed in the LC–MS spectrum. The minor impurity could not be removed after several chromatographic purifications. Further attempts for the synthesis or purification of this impurity could not be undertaken due to the lack of material. In spite of the presence of the impurity, the synthetic *O*-acetyltalpinine **11** was fully characterized and the structural assignments could be carried out to confirm the synthesis unambiguously by high resolution NMR spectroscopy.)

### 3.6. N_4_-Methyltalpinine (***12***) as the Chloride Salt

(Preparation of the HCl solution for NMR titration: anhydrous HCl (0.3 mL, 4.0 M solution in dioxane) was dissolved in 5.0 mL of dry CDCl_3_. The solution, which resulted, was gradually added via a micropipette into the reaction vessel.)

The indole **9** (1.0 mg, 0.003 mmol) was dissolved in dry CDCl_3_ (1.0 mL) in an oven-dried NMR tube (5 mm OD). The above HCl solution (25 μL in total) was gradually added to the NMR tube via a micropipette. The reaction that resulted was kept at room temperature for 2 h. After that, examination of the ^1^H NMR spectrum indicated complete conversion of the aldehyde into the desired product. The solvent was removed under reduced pressure to afford *N*_4_-methyltalpinine **12** as a chloride salt (1.1 mg, 99%), a colorless solid. This residue was used for characterization without any purification. The optical rotation and spectroscopic data for the synthetic *N*_4_-methyltalpinine **12** were in excellent agreement with the values reported in the literature for the natural product [23] by Kinghorn et al. For ^1^H NMR results: see Table 5; for ^13^C NMR: see Table 5; [α]_D_^25^: Synthetic = -9.1 (c 0.11, EtOH); Natural [23]: = −10 (c 0.1, MeOH); HRMS: (ESI) *m/z* M^+^ calculated for C_21_H_27_N_2_O_2_ 339.2067, found 339.2036.

## 4. Conclusions

In summary, the first total synthesis of several C-19 methyl-substituted bioactive indole alkaloids has been successfully completed. Macrocarpines F (**6**) and G (**7**) and bioactive alkaloid *O*-acetyltalpinine (**11**), as well as the potent NF-κB inhibitor *N*_4_-methyltalpinine (**12**) as a chloride salt, have been synthesized for the first time. The other bioactive alkaloid, talpinine (**10**), had been previously synthesized by Yu et al. [34], but the strategy developed here is shorter and gives a higher yield. The previously reported ambidextrous Pictet–Spengler/Dieckmann protocol and subsequent copper-mediated cross-coupling process provided improved access to the sarpagine/macroline scaffold, containing the stereogenic methyl function at the biogenetic C-19 position. This work provides a proof of concept for the total synthesis of *N*_b_-H-containing C-19 methyl-substituted sarpagine/macroline alkaloids utilizing a general synthetic approach. In addition, the acid-mediated quaternary hemiaminal formation should provide efficient and rapid access to the important *N*_4_-methyltalpinine and related alkaloids. Work on the synthesis of other alkaloids from this group, as well as the total synthesis of the unnatural enantiomer of these alkaloids, is underway and will be reported in due course.

## Figures and Tables

**Table 1:** Comparison of the ^1^H and ^13^C NMR of natural [20] and synthetic macrocarpine F (**6**) in CDCl_3_.

H#	^1^H NMR Natural (400 MHz) [20]	^1^H NMR Synthetic (300 MHz)	C#	^13^C NMR Natural (100 MHz) [20]	^13^C NMR Synthetic (75 MHz)
3	4.27 (m)	4.37 (br s)	2	136.4	136.3
5	3.21 (m)	3.23–3.15 (m)	3	46.5	46.5
6β 6α	2.63 (d, *J* = 15 Hz) 3.17 (m)	2.68 (br d, *J* = 14.7 Hz) 3.30–3.23 (m)	5	48.2	48.2
9	7.46 (br d, *J* = 8 Hz)	7.49 (d, *J* = 7.6 Hz)	6	28.5	28.4
10	7.07 (br t, *J* = 8 Hz)	7.14–7.06 (m)	7	107.7	107.9
11	7.18 (br t, *J* = 8 Hz)	7.23–7.16 (m)	8	126.6	126.2
12	7.75 (br d, *J* = 8 Hz) *	7.32–7.23 (d, *J* = 8.1 Hz) ^#^	9	118.1	118.1
14β 14α	1.35 (m) 2.45 (td, *J* = 12, 4 Hz)	1.31–1.25 (m) ^$^ 1.46–1.35 (m)	10	119.1	119.0
15	2.10 (m)	2.19–2.11 (m) ^$^	11	121.2	121.1
16	2.08 (m)	2.28–2.12 (m) ^$^	12	108.9	108.8
17β 17α	3.78 (dd, *J* = 11, 5 Hz) 4.07 (t, *J* = 11 Hz)	3.88–3.79 (m) 4.19–4.08 (t, *J* = 11.6 Hz)	13	136.8	- ^§^
19	3.94 (qd, J = 6.8, 2 Hz)	4.02–3.90 (m)	14	29.8	29.7
20	1.04 (m)	1.13–1.01 (m)	15	29.0	29.5
21	3.65 (dd, *J* = 11, 4 Hz)	3.75–3.65 (m)	16	38.1	38.3
21′	3.72 (dd, *J* = 11, 6 Hz)	3.88–3.79 (m) ^$^	17	68.6	68.6
18-Me	1.21 (d, *J* = 6.8 Hz)	1.29–1.19 (m) ^$^	18	18.9	18.8
*N*(1)-Me	3.52 (s)	3.61 (s)	19	71.3	71.4
			20	44.1	43.9
			21	62.5	63.1
			*N*(1)-Me	29.0	29.0

* There is a typographical error in the natural [20] macrocarpine F at H-12. ^#^ Merged with the chloroform peak; confirmed by COSY and HSQC NMRs. ^$^ Overlapped peaks. ^§^ The quaternary carbon (C-13) atom was not visible in the ^13^C NMR spectrum at this concentration.

**Table 2:** Comparison of the ^1^H NMR of natural [20] and synthetic macrocarpine G (**7**) in CDCl_3_.

H#	^1^H NMR Natural (400 MHz) [20]	^1^H NMR Synthetic (500 MHz)
3	4.27 (br t, *J* = 3 Hz)	4.35 (br s)
5	3.25 (m)	3.31 (d, *J* = 7.3 Hz)
6β 6α	2.58 (d, *J* = 16 Hz) 3.18 (dd, *J* = 16, 7 Hz)	2.65 (br d, *J* = 16.2 Hz) 3.25-3.19 (m)
9	7.46 (br d, *J* = 7.5 Hz)	7.49 (d, *J* = 7.9 Hz)
10	7.08 (td, *J* = 7.5, 1 Hz)	7.10 (t, *J* = 7.5 Hz)
11	7.17 (td, *J* = 7.5, 1 Hz)	7.19 (t, *J* = 7.5 Hz)
12	7.24 (br d, *J* = 7.5 Hz)	7.29 (d, *J* = 8.1 Hz)
14β 14α	1.49 (dt, *J* = 12, 2 Hz) 2.18 (td, *J* = 12, 4 Hz)	1.58-1.53 (m) * 2.28 (td, *J* = 12.6, 3.8 Hz)
15	2.02 (m)	2.15–2.08 (m)
16	1.81 (dt, *J* = 12, 5 Hz)	1.91–1.86 (m) *
17β 17α	3.72 (dd, *J* = 12, 4 Hz) 4.03 (t, *J* = 12 Hz)	3.78 (dd, *J* = 11.4, 4.4 Hz) 4.10 (br t, *J* = 12.2 Hz)
19	3.46 (m)	3.58–3.54 (m) *
20	1.37 (m)	1.52–1.45 (m) *
21	3.22 (m)	3.39–3.34 (m)
21′	3.40 (dd, *J* = 11, 5 Hz)	3.51 (dd, *J* = 11.0, 5.3 Hz) *
18-Me	1.11 (d, *J* = 6 Hz)	1.17 (d, 6.1 Hz)
*N*(1)-Me	3.52 (s)	3.63 (s)

* Overlapped peaks.

**Table 3:** Comparison of the ^1^H and ^13^C NMR spectra of synthetic talpinine (**10**) and the previous synthesis by Yu et al. [34] with CDCl_3_.

H#	^1^H NMR Synthetic (400 MHz)	^1^H NMR Synthetic (300 MHz) Yu et al. [34]	C#	^13^C NMR Synthetic (75 MHz)	^13^C NMR Synthetic (75 MHz) Yu et al. [34]
3	4.43 (d, *J* = 7.9 Hz)	4.39 (d, *J* = 12.1 Hz)	2	139.1	138.48
5	3.52–3.41 (m) ^$^	3.50 (t, *J* = 6.3 Hz)	3	40.2	40.51
6β 6α	2.65 (d, *J* = 15.6 Hz) 3.21 (dd, *J* = 15.6, 6.0 Hz)	2.60 (d, *J* = 15.6 Hz) 3.20 (dd, *J* = 15.8, 5.8 Hz)	5	49.8	50.18
9	7.47 (m)	7.45 (d, *J* = 7.7 Hz)	6	26.3	26.63
10	7.12–7.05 (m)	7.07 (t, *J* = 7.9 Hz)	7	103.3	103.55
11	7.19 (t, *J* = 7.7 Hz)	7.17 (t, *J* = 7.8 Hz)	8	127.3	127.50
12	7.31–7.27 (m)	7.27 (d, *J* = 8.0 Hz)	9	118.3	118.69
14β 14α	1.38–1.20 (m) * 1.87–1.79 (m)	1.30 (d, *J* = 12.1 Hz) 1.81 (t, *J* = 12.0 Hz)	10	118.9	119.28
15	1.93–1.86 (m)	1.87 (d, *J* = 3.0 Hz)	11	120.9	121.31
16	1.38–1.20 (m) *	1.20 (m)	12	108.7	109.12
17β 17α	3.52–3.41 (m) ^$^ 3.72–3.62 (m)	3.42 (d, *J* = 11.0 Hz) 3.65 (t, *J* = 11.4 Hz)	13	137.5	137.73
19	4.10–3.99 (m)	4.03 (q, *J* = 6.8 Hz)	14	31.9	32.31
20	1.38–1.20 (m) *	1.25 (m)	15	23.3	23.63
21	4.71 (m)	4.69 (d, *J* = 1.8 Hz)	16	35.2	35.58
18-Me	1.38–1.20 (m) *	1.27 (d, *J* = 6.0 Hz)	17	64.0	64.35
*N*(1)-Me	3.59 (s)	3.55 (s)	18	15.7	16.10
			19	72.6	72.98
			20	43.6	44.13
			21	87.9	88.25
			*N*(1)-Me	29.2	29.63

* and ^$^ Overlapped peaks.

**Table 4:** Comparison of the ^1^H and ^13^C NMR spectra of natural [20] and synthetic *O*-acetyltalpinine (**11**) in CDCl_3_.

H#	^1^H NMR Natural (400 MHz) [20]	^1^H NMR Synthetic (500 MHz)	C#	^13^C NMR Natural (100 MHz) [20]	^13^C NMR Synthetic (125 MHz)
3	4.48 (br dd, *J* = 10, 2 Hz)	4.46 (br d, *J* = 9.3 Hz)	2	138.9	- *
5	3.52 (br t, *J* = 5.5 Hz)	3.56 (br t, *J* = 5.5 Hz)	3	41.7	41.7
6β 6α	2.66 (d, *J* = 15.6 Hz) 3.20 (dd, *J* = 15.6, 5.5 Hz)	2.62 (br d, *J* = 15.8 Hz) 3.18 (dd, *J* = 15.2, 5.9 Hz)	5	50.3	50.3
9	7.47 (br d, *J* = 7.5 Hz)	7.47 (d, *J* = 7.7 Hz)	6	26.6	26.6
10	7.09 (br td, *J* = 7.5 Hz)	7.09 (t, *J* = 7.4 Hz)	7	103.1	103.1
11	7.19 (td, *J* = 7.5, 1 Hz)	7.18 (t, *J* = 7.6 Hz)	8	127.2	127.2
12	7.29 (br d, *J* = 7.5 Hz)	7.29 (d, *J* = 8.2 Hz)	9	118.1	118.1
14β 14α	1.52 (ddd, *J* = 12, 4, 2.8 Hz) 1.89 (ddd, *J* = 12, 10, 1.6 Hz)	1.53–1.47 (m) 1.91–1.83 (m) *	10	120.9	120.9
15	2.00 (m)	2.03–1.97 (m)	11	118.8	118.9
16	1.30 (m)	1.30 (m) *	12	108.7	108.7
17β 17α	3.47 (dd, *J* = 11, 2 Hz) 3.71 (dd, *J* = 11, 1 Hz)	3.47 (dd, *J* = 11.4, 1.9 Hz) 3.71 (dd, *J* = 11.4, 1.1 Hz)	13	137.4	137.4
19	4.34 (q, *J* = 7 Hz)	4.34 (q, *J* = 7.0 Hz)	14	32.3	32.3
20	1.34 (m)	1.34–1.31 (m) *	15	23.2	23.2
21	5.63 (br d, *J* = 2 Hz)	5.61 (m)	16	35.2	35.2
18-Me	1.30 (d, *J* = 7 Hz)	1.30 (d, 6.8 Hz)	17	63.8	63.8
*N*(1)-Me	3.66 (s)	3.66 (s)	18	15.6	15.7
			19	71.6	71.6
			20	43.6	43.6
			21	88.9	88.9
			*N*(1)-Me	29.3	29.3
			*21*-OAc	21.2	21.3
			*21*-OAc	169.6	169.6

* Overlapped peaks; the peak for C-2 did not show up in the ^13^C NMR spectrum at this concentration.

**Table 5:** Comparison of the ^1^H and ^13^C NMR spectra of synthetic and natural [23] *N*_4_-metyltalpinine **12** in CD_3_OD.

H#	^1^H NMR Natural (400 MHz) [23]	^1^H NMR Synthetic (500 MHz)	C#	^13^C NMR Natural (100 MHz) [23]	^13^C NMR Synthetic (125 MHz)
3	4.99 (1H, d, *J* = 10.7 Hz)	4.99 (1H, d, *J* = 10.4 Hz)	2	134.2	134.2
5	3.91 (1H, t, *J* = 5.4 Hz)	3.92 (1H, t, *J* = 5.5 Hz)	3	53.1	53.1
6β 6α	3.09 (1H, d, *J* = 16.6 Hz) 3.36 (1H, dd, *J* = 17.4, 5.3 Hz)	3.09 (1H, d, *J* = 17.4 Hz) 3.36 (1H, dd, *J* = 17.2, 5.4 Hz)	5	61.3	61.3
9	7.53 (1H, d, *J* = 7.9 Hz)	7.53 (1H, d, *J* = 7.9 Hz)	6	24.7	24.7
10	7.12 (1H, t, *J* = 7.8 Hz)	7.12 (1H, t, *J* = 7.6 Hz)	7	101.2	101.6
11	7.25 (1H, t, *J* = 7.7 Hz)	7.25 (1H, t, *J* = 7.7 Hz)	8	127.3	127.3
12	7.43 (1H, d, *J* = 8.2 Hz)	7.43 (1H, d, *J* = 8.3 Hz)	9	119.5	119.5
14β 14α	1.98 (1H, ddd, *J* = 13.2, 5.0, 1.8 Hz) 2.47 (1H, br t, *J* = 12.2 Hz)	1.96 (1H, ddd, *J* = 13.3, 5.0, 1.8 Hz) 2.47 (1H, m)	10	121.0	121.0
15	2.36 (1H, br s)	2.36 (1H, m)	11	123.7	123.7
16	1.77 (1H, br s)	1.77 (1H, br s)	12	110.6	110.6
17β 17α	3.80 (1H, d, *J* = 11.7 Hz) 3.54 (1H, dd, *J* = 11.9, 2.1 Hz)	3.80 (1H, d, *J* = 11.7 Hz) 3.54 (1H, dd, *J* = 11.9, 2.2 Hz)	13	139.7	139.7
19	4.15 (1H, q, *J* = 6.8 Hz)	4.15 (1H, q, *J* = 6.9 Hz)	14	32.4	32.3
20	2.04 (1H, br s)	2.04 (1H, br s)	15	22.7	22.6
21	4.95 (1H, d, *J* = 1.9 Hz)	4.95 (1H, d, *J* = 1.7 Hz)	16	38.2	38.2
18-Me	1.38 (3H, d, *J* = 6.8 Hz)	1.38 (3H, d, *J* = 6.8 Hz)	17	63.1	63.1
*N*(1)-Me	3.73 (3H, s)	3.73 (3H, s)	18	15.7	15.7
*N*(4)-Me	3.07 (3H, s)	3.07 (3H, s)	19	72.7	72.7
			20	48.2	48.2
			21	98.4	98.4
			*N*(1)-Me	29.9	29.9
			*N*(4)-Me	43.5	43.5

## Data Availability

The data presented in this study are available on request from the corresponding author.

## Supplementary Materials

The following are available online, 1-D and 2-D NMR Spectra of alkaloids **6**, **7**, **10**, **11**, and **12**.
