# Supplementary material for: Completion of the Total Synthesis of Several Bioactive Sarpagine/Macroline Alkaloids including the Important NF-κB Inhibitor N4-Methyltalpinine
Source: Molecules. 2022 Mar 7;27(5):1738. doi: 10.3390/molecules27051738 (PMC8911669; doi:10.3390/molecules27051738)
Supplement: Supplementary file 1 [file molecules-27-01738-s001.zip › molecules-1599387-supplementary.pdf]

Supporting Information

Full Paper

Completion of the Total Synthesis of Several Bioactive Sarpagine/Macroline Alkaloids Including the Important NF-κB Inhibitor N_4-Methyltalpinine[#,§]

Md Toufiqur Rahman, V.V.N. Phani Babu Tiruveedhula, Michael Rajesh Stephen, Sundari K. Rallapalli, Kamal P. Pandey, and James M. Cook*

Department of Chemistry & Biochemistry, University of Wisconsin-Milwaukee, Milwaukee, WI-53211
*Corresponding Author Email: capncook@uwm.edu

[#]This article is dedicated to Professor Daniel Comins for his outstanding contributions to Heterocyclic Chemistry and science as a whole.

[§]Taken in Parts from MTR's Doctoral Dissertation

Section	Pages
1-D and 2-D NMR Spectra of alkaloids **6**,**7**,**10**,**11**, and **12**	2-23

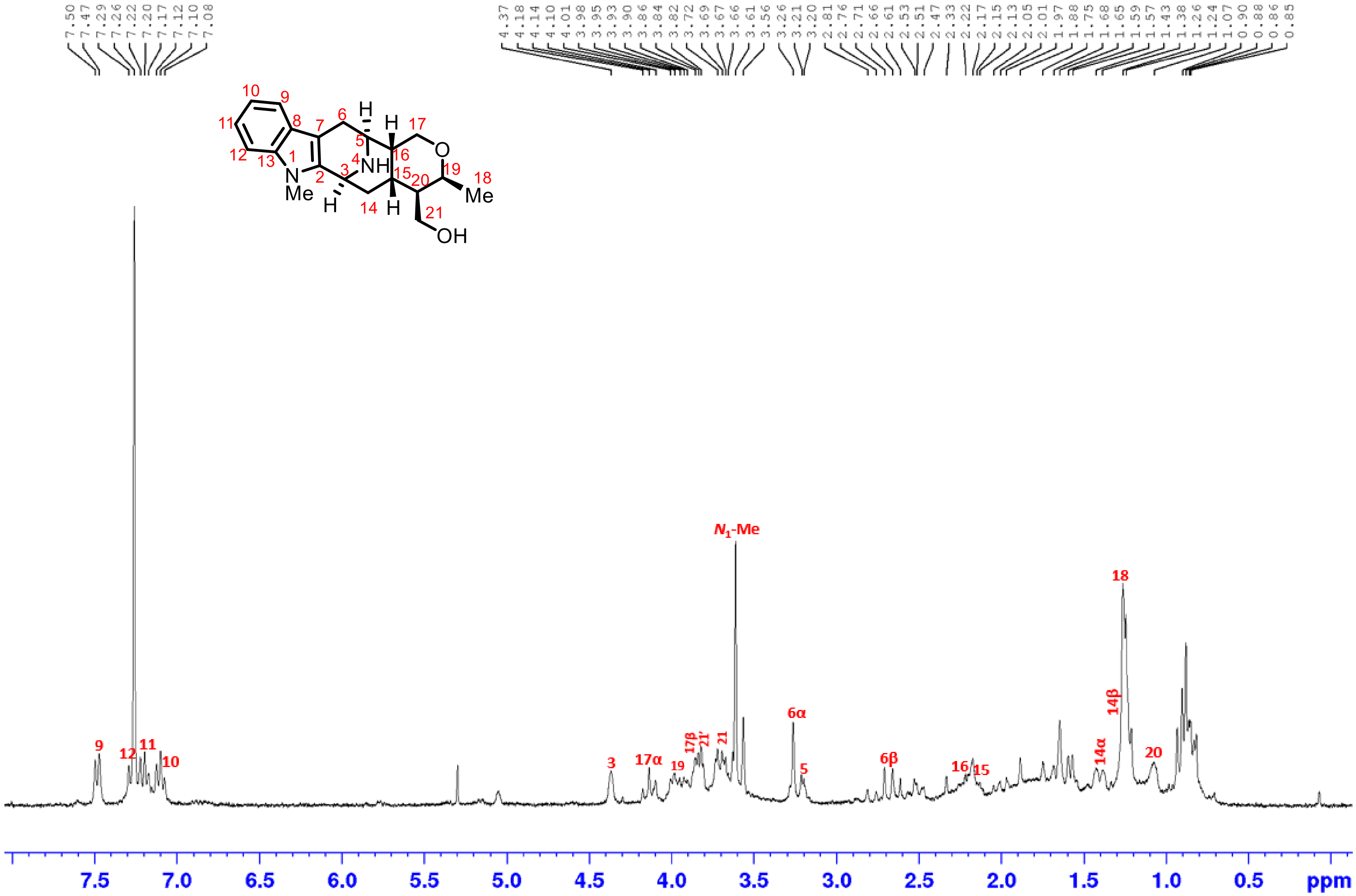

^{1}H NMR spectrum of macrocarpine F **6** (300 MHz, $CDCl_3$); Assignment is based on ^{1}H, DEPT-135, COSY and HSQC NMR correlations

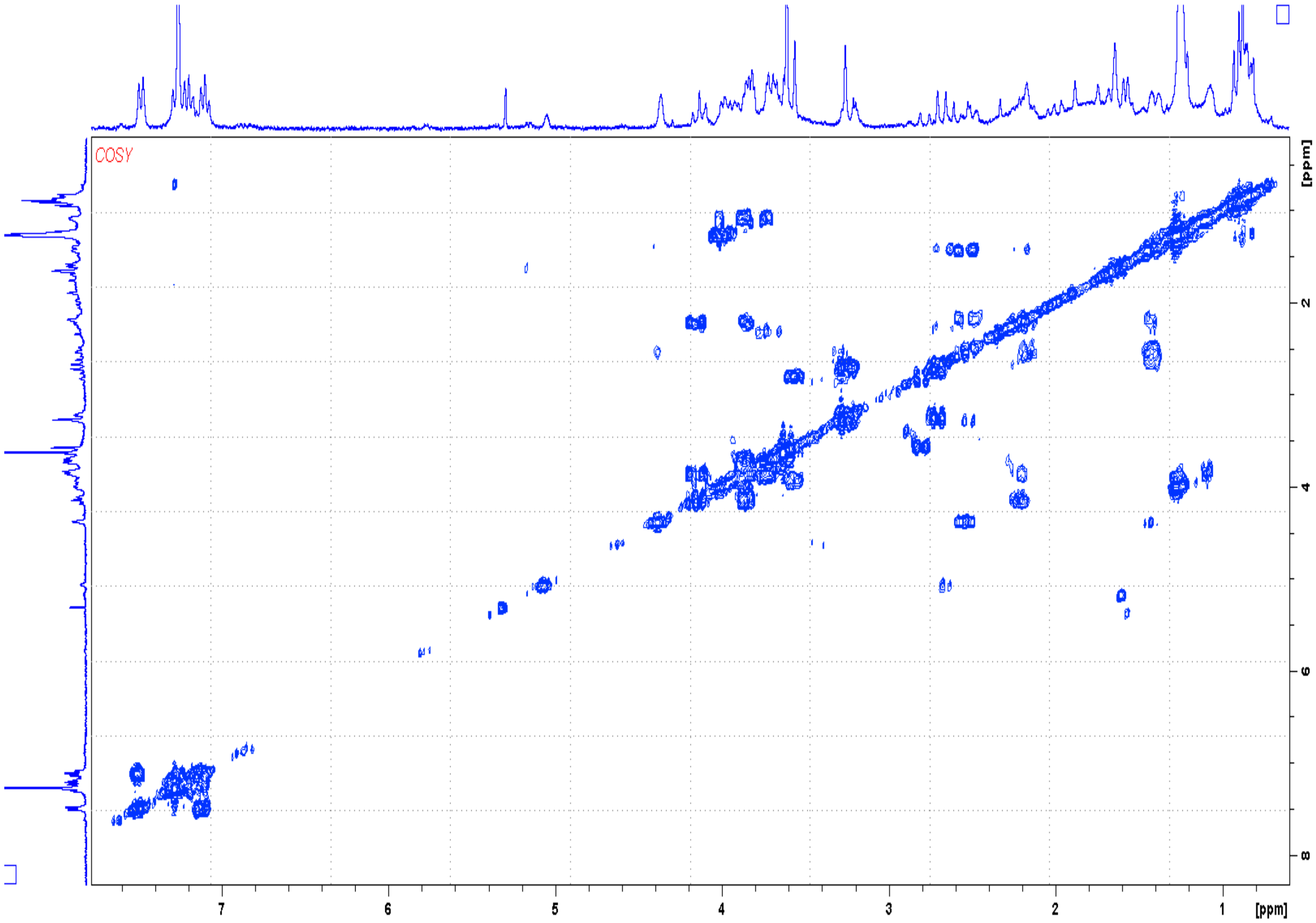

COSY NMR spectrum of macrocarpine F **6** (300 MHz, $CDCl_3$)

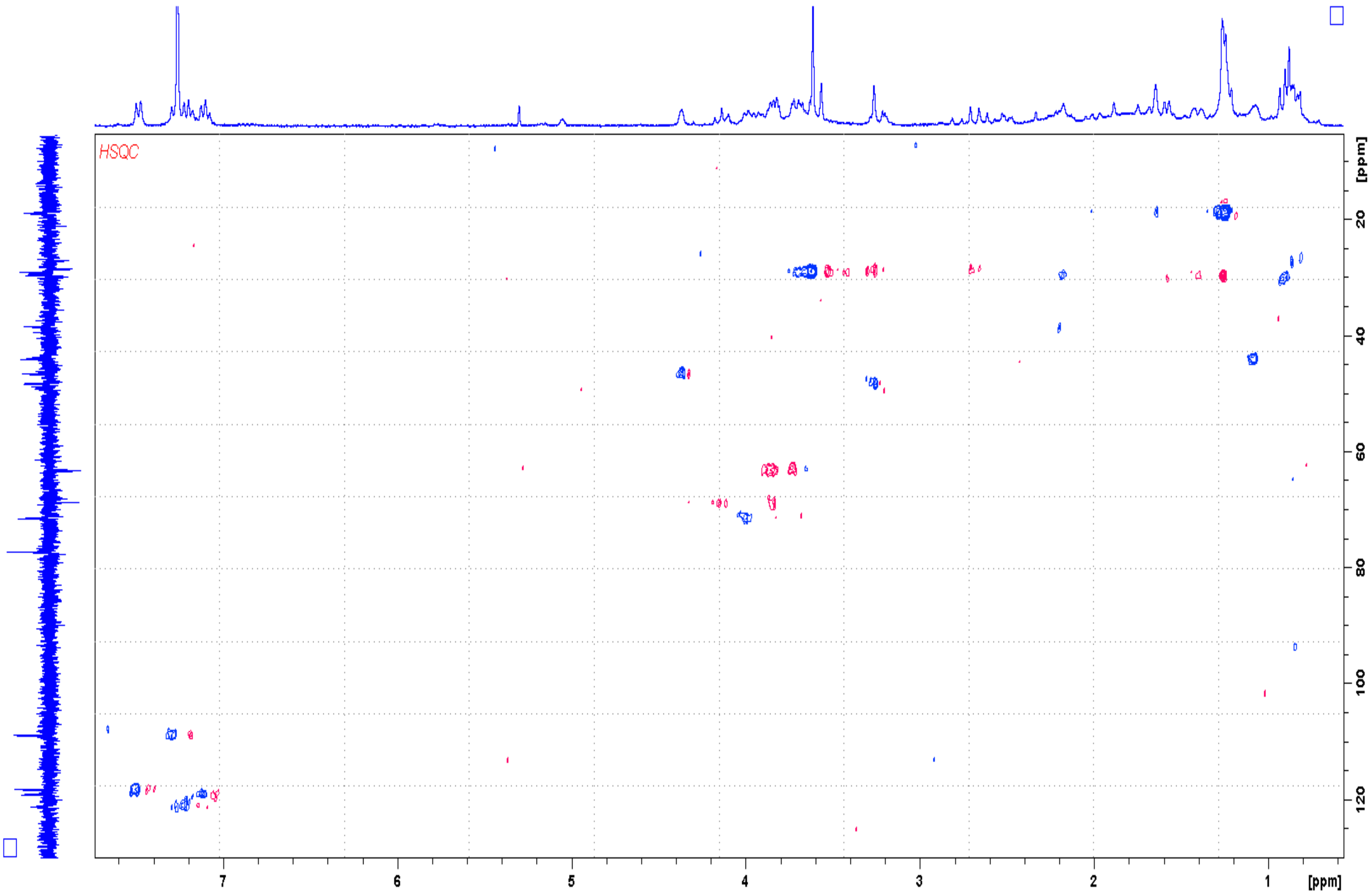

HSQC NMR spectrum of macrocarpine F **6** (300, 75 MHz, $CDCl_3$)

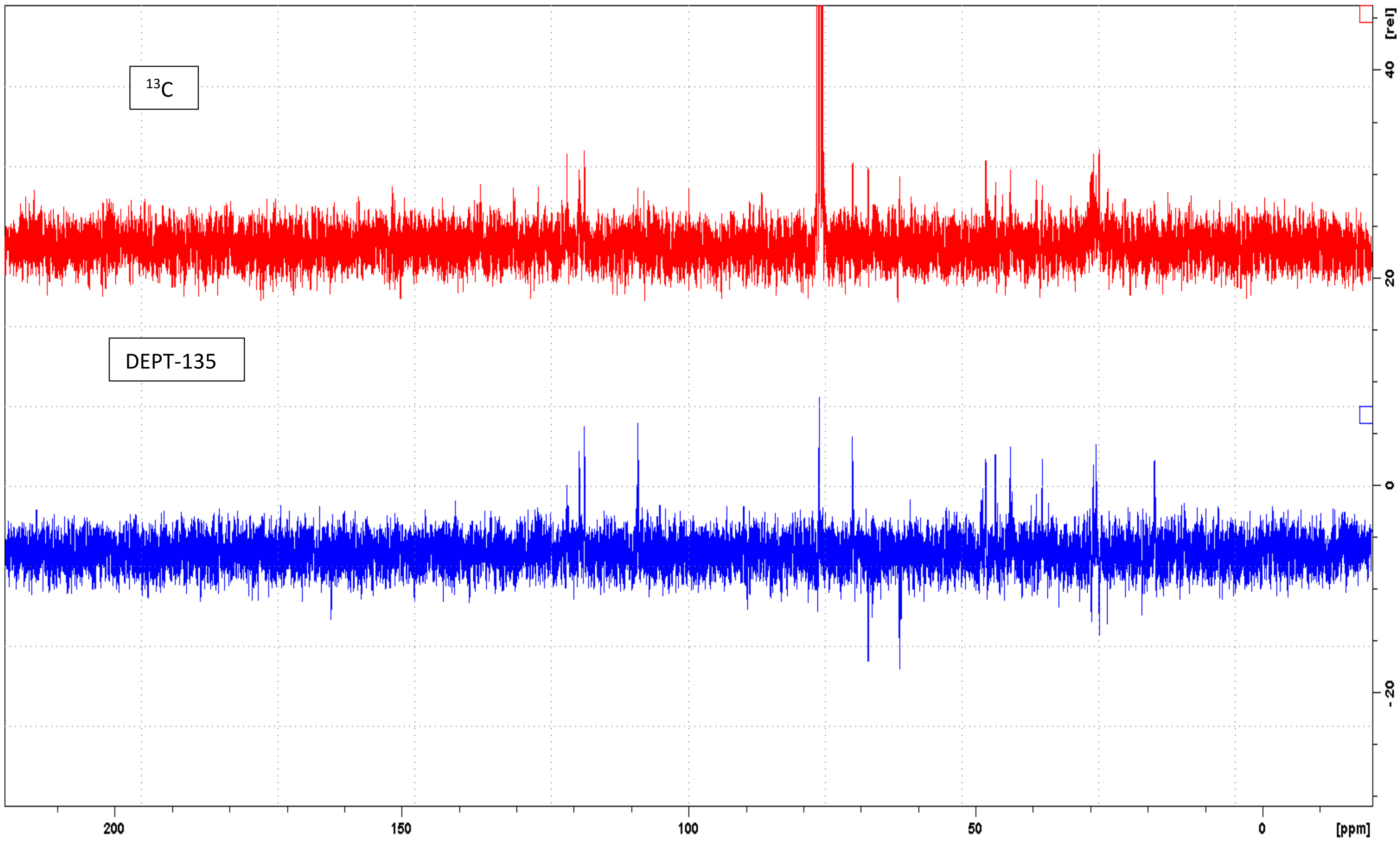

^{13}C and DEPT-135 NMR spectra of macrocarpine F **6** (75 MHz, $CDCl_3$)

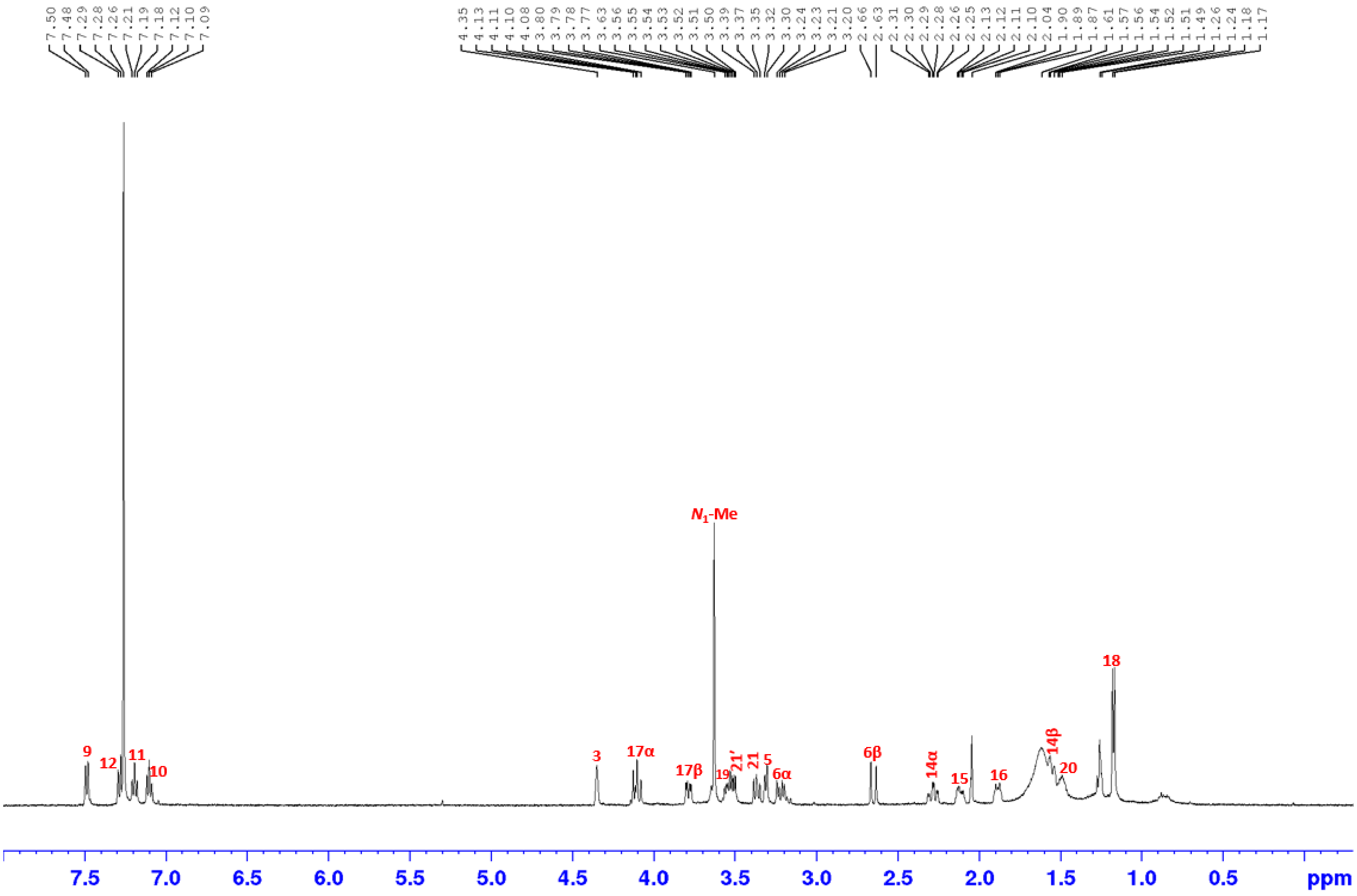

^{1}H NMR spectrum of synthetic macrocarpine G **7** (500 MHz, $CDCl_3$)

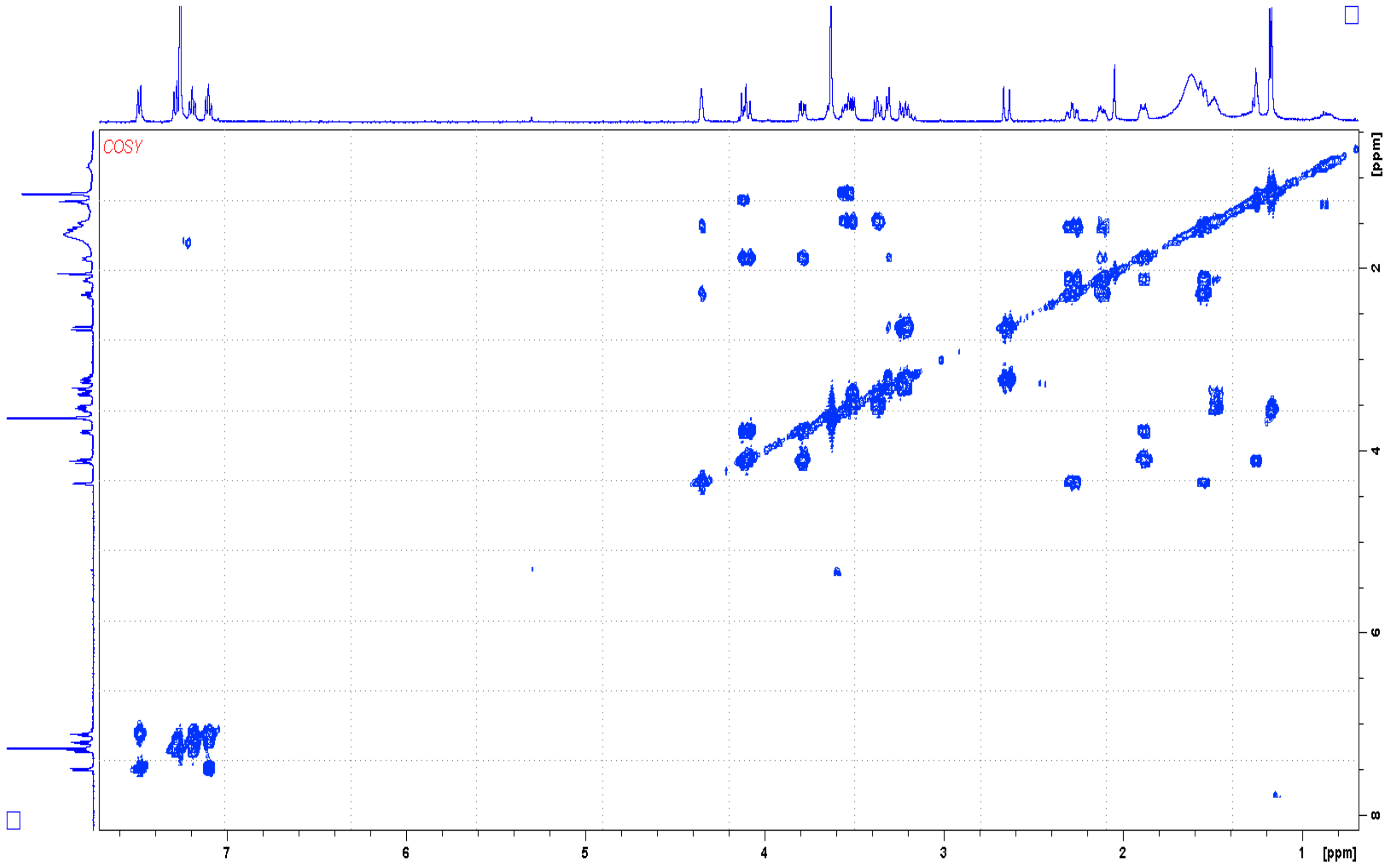

COSY NMR spectrum of synthetic macrocarpine G **7** (500 MHz, $CDCl_3$)

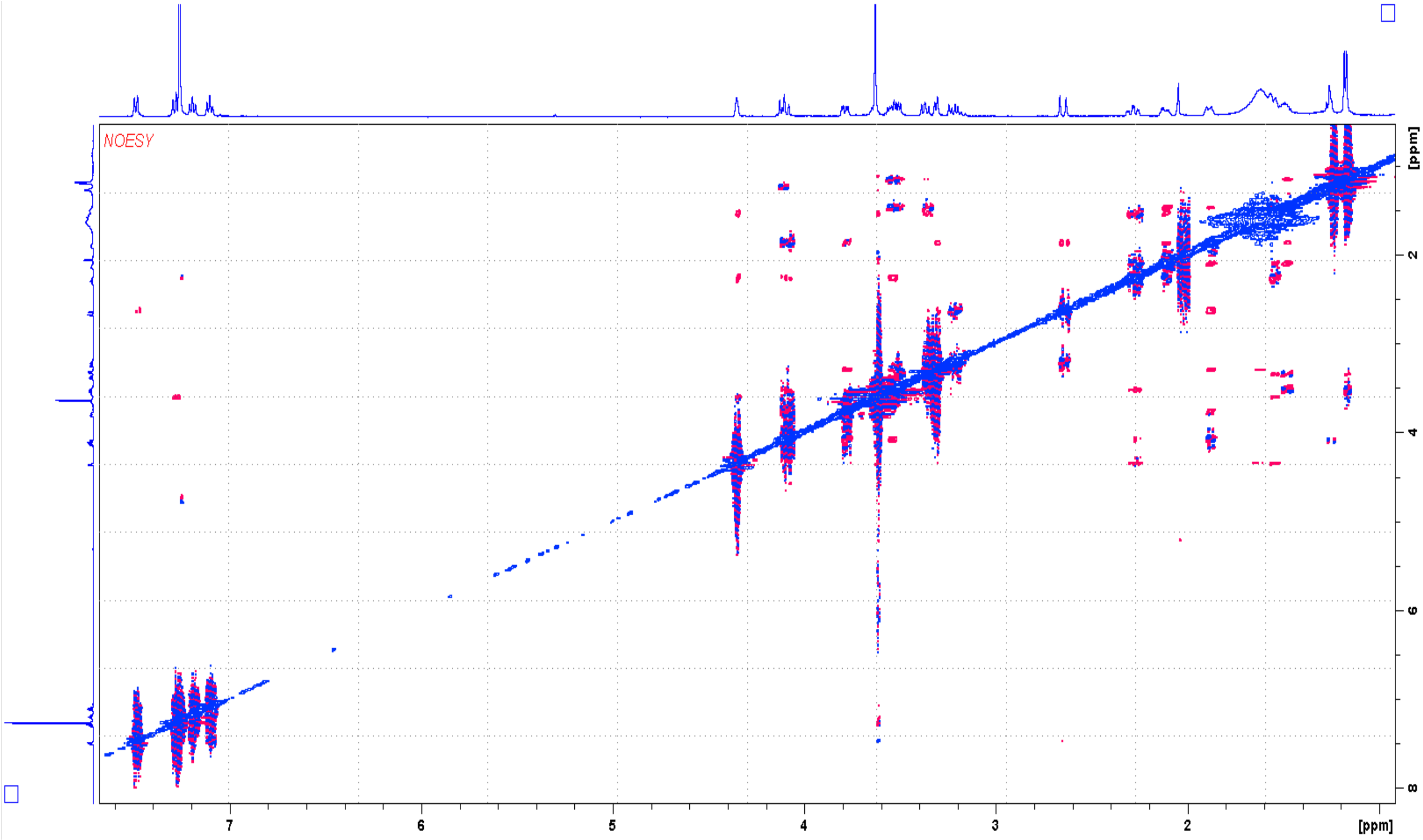

NOESY NMR spectrum of macrocarpine G **7** (500 MHz, $CDCl_3$)

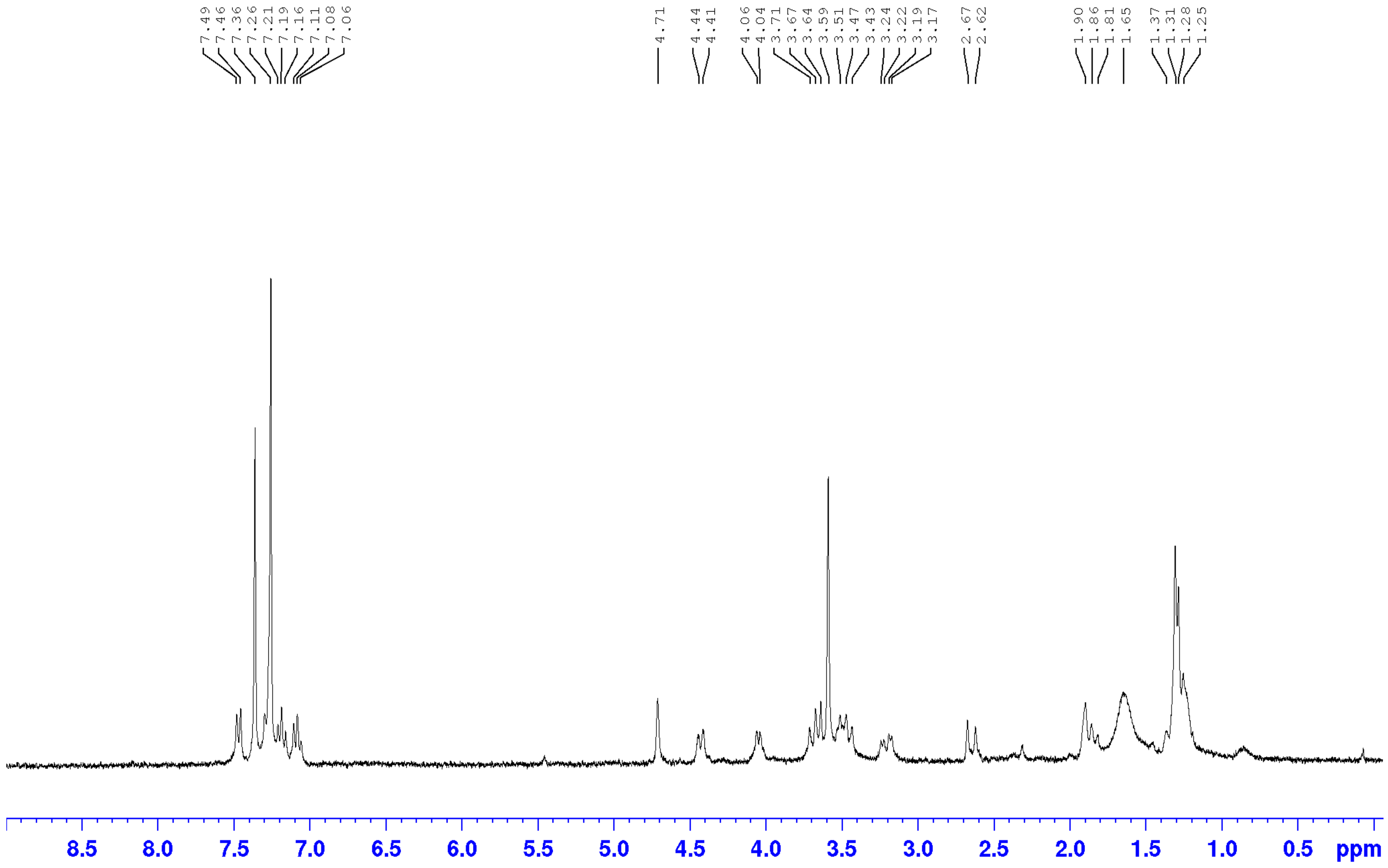

^{1}H NMR spectrum of talpinine **10** (300 MHz, $CDCl_3$)

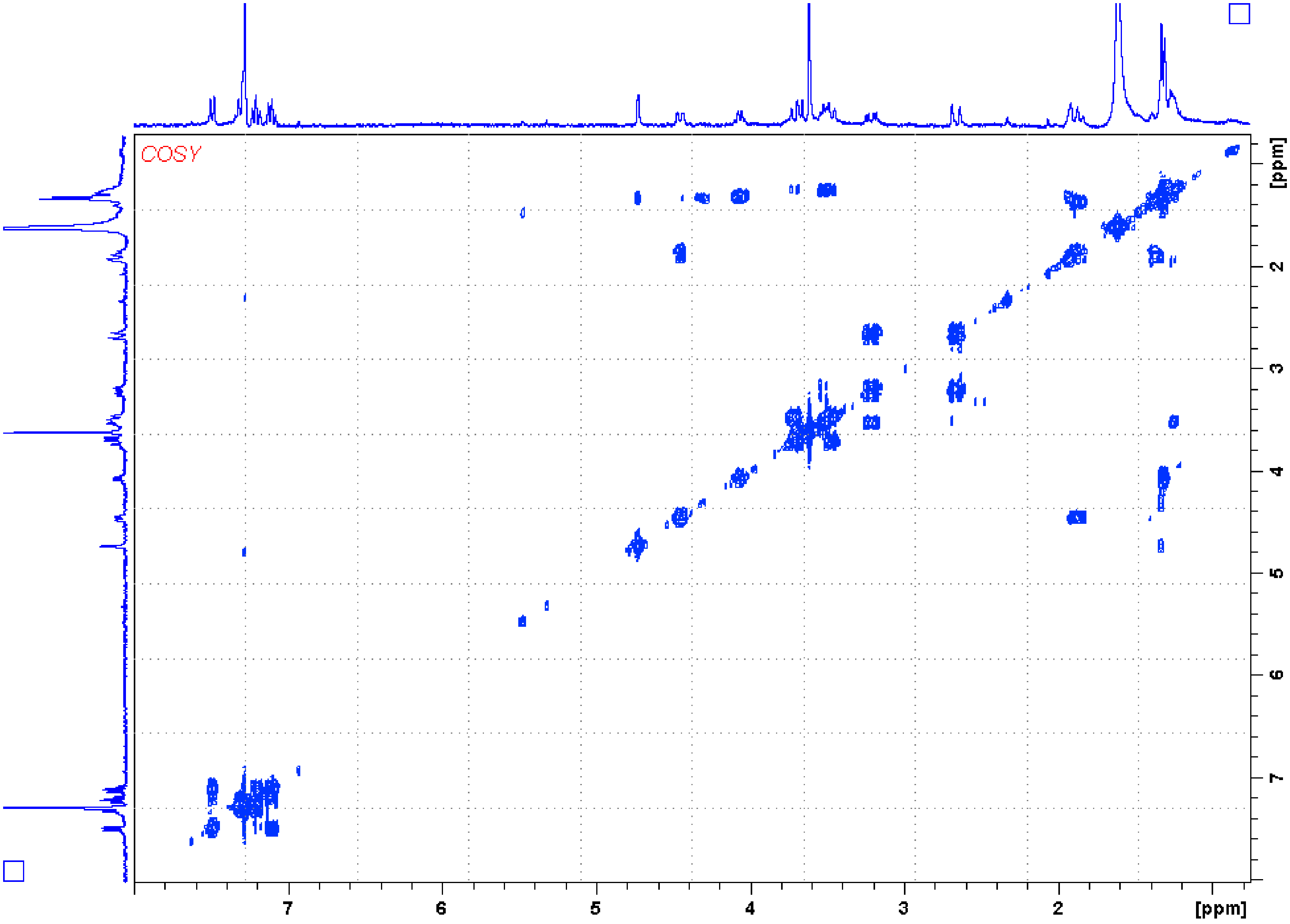

COSY NMR spectrum of talpinine **10** (300 MHz, $CDCl_3$)

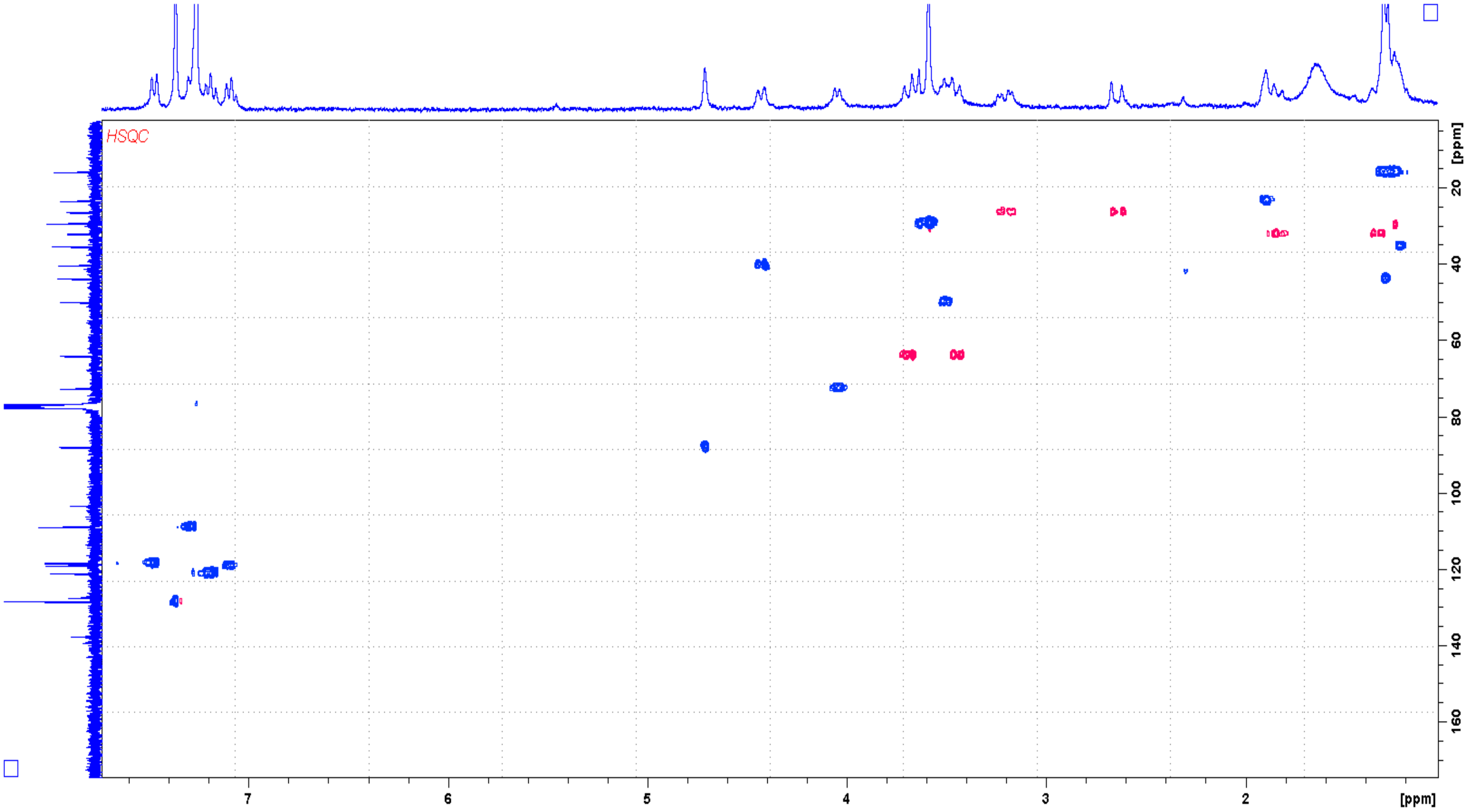

HSQC NMR spectrum of talpinine **10** (300, 75 MHz, $CDCl_3$)

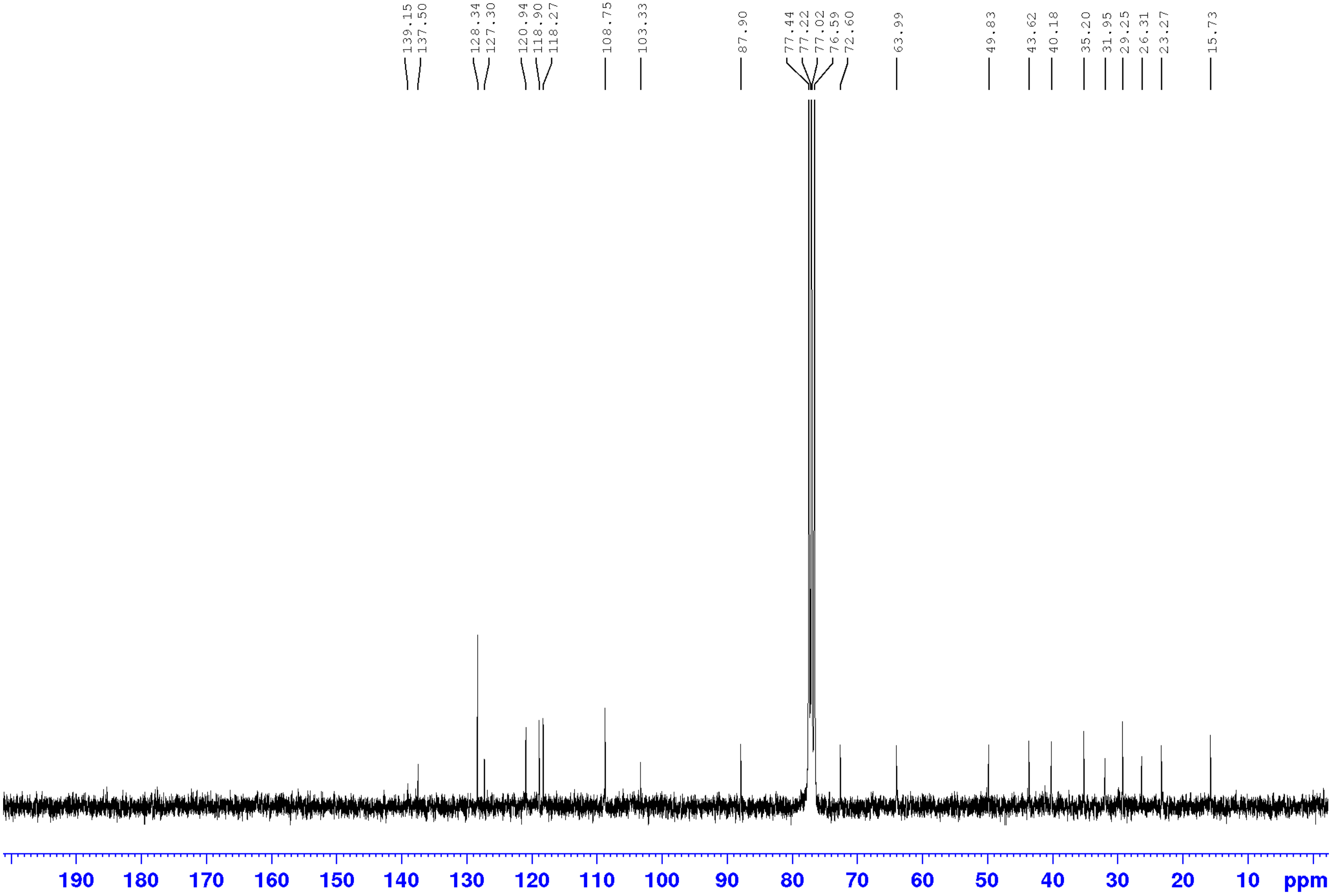

^{13}C NMR spectrum of talpinine **10** (75 MHz, $CDCl_3$)

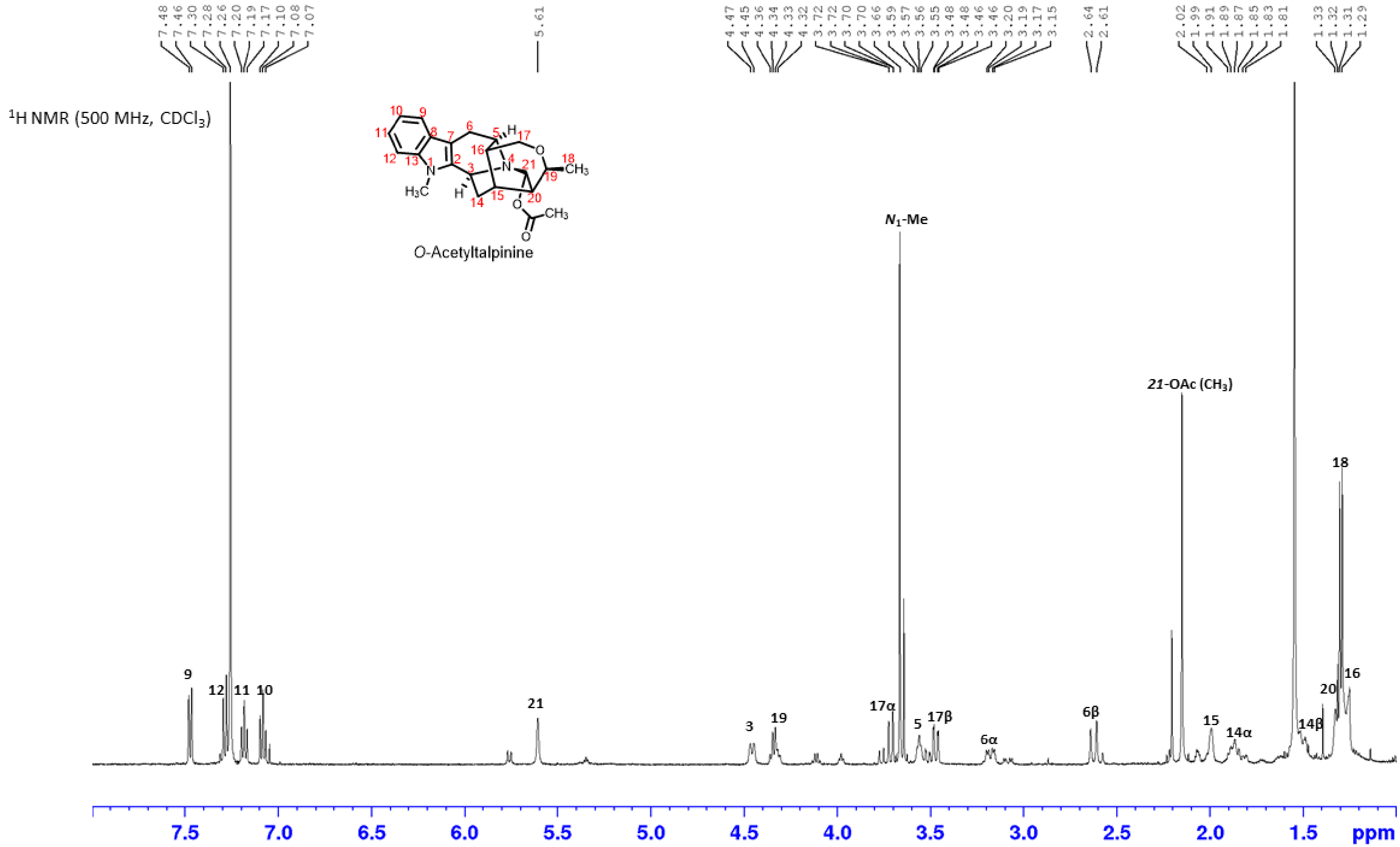

Assigned ^{1}H spectrum of *O*-acetyltalpinine **11** (500 MHz, $CDCl_3$). Assignment was based on ^{1}H, ^{13}C and HSQC NMR correlations

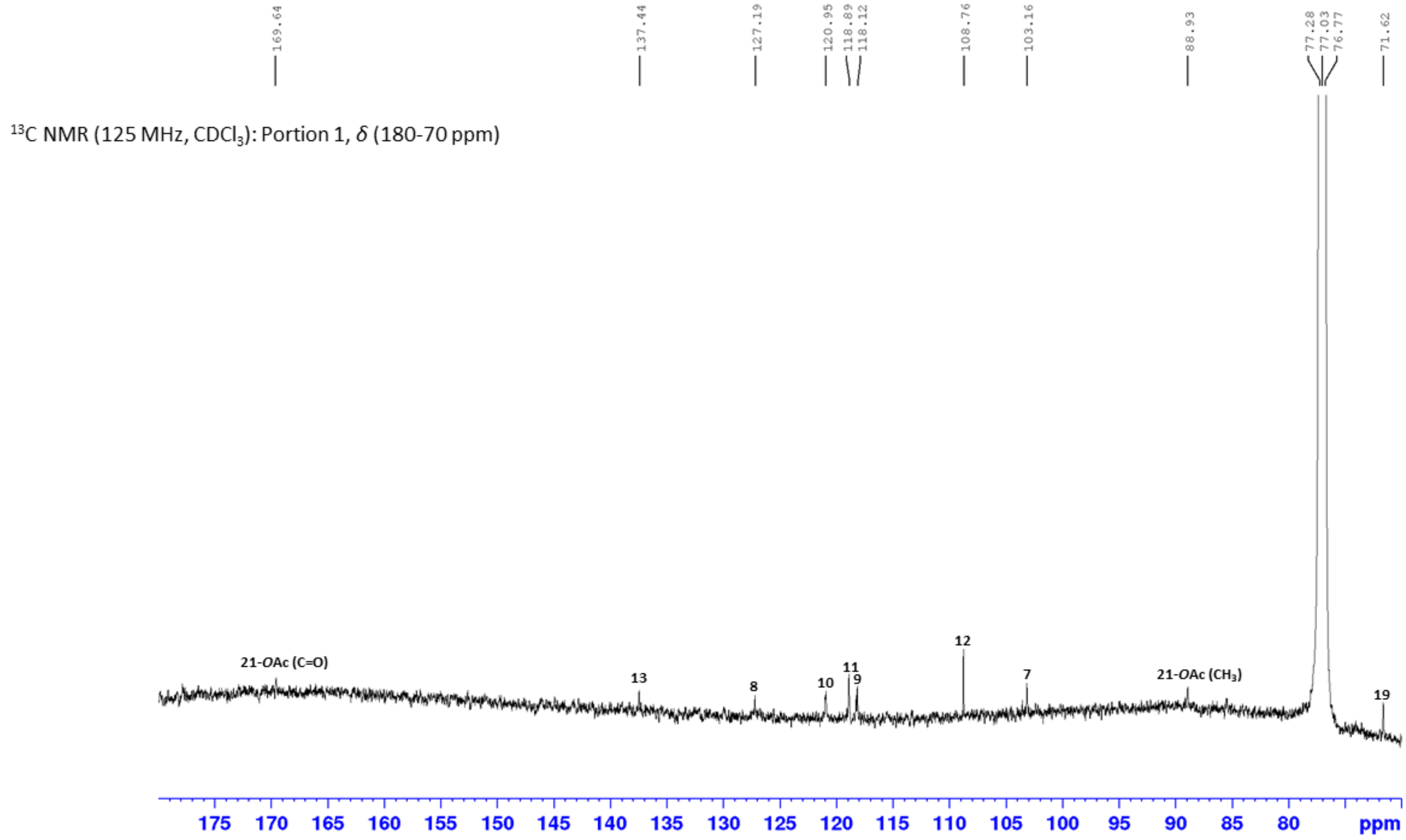

^{13}C NMR spectrum δ 180-70 ppm (125 MHz, $CDCl_3$) of *O*-acetyltalpinine **11** (Assignment is based on ^{1}H, ^{13}C and HSQC NMR correlations)

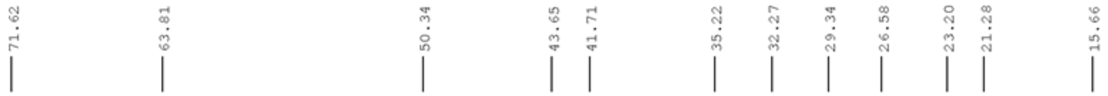

^{13}C NMR (125 MHz, $CDCl_3$): Portion 2, δ (75-0 ppm)

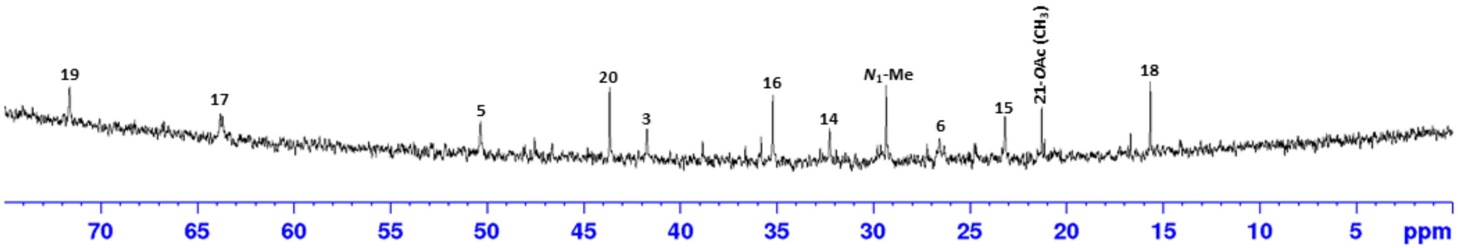

^{13}C NMR spectrum δ 75-0 ppm (125 MHz, $CDCl_3$) of *O*-acetyltalpinine **11**

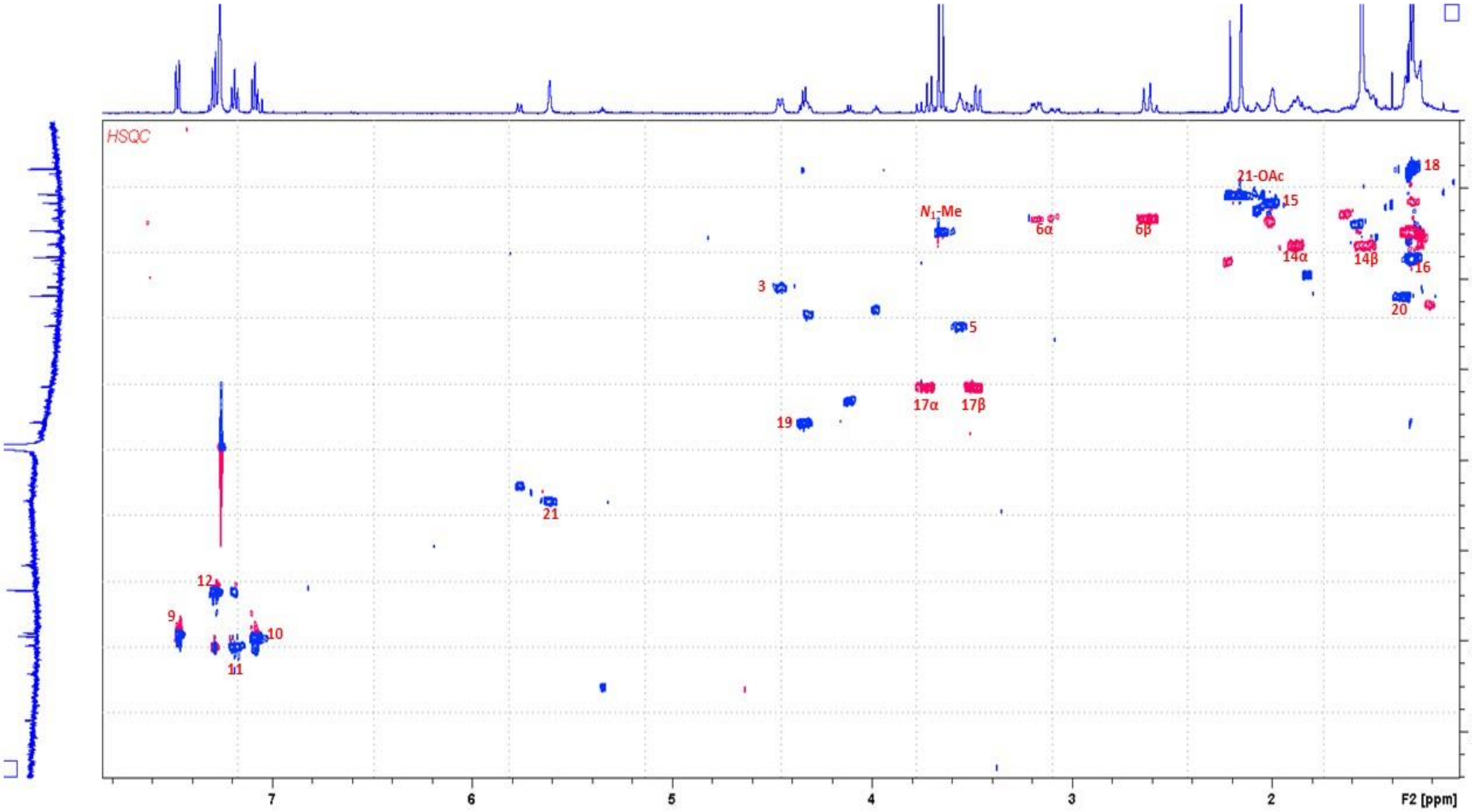

Assigned HSQC NMR spectrum of *O*-Acetyltalpinine **11** (500, 125 MHz, $CDCl_3$)

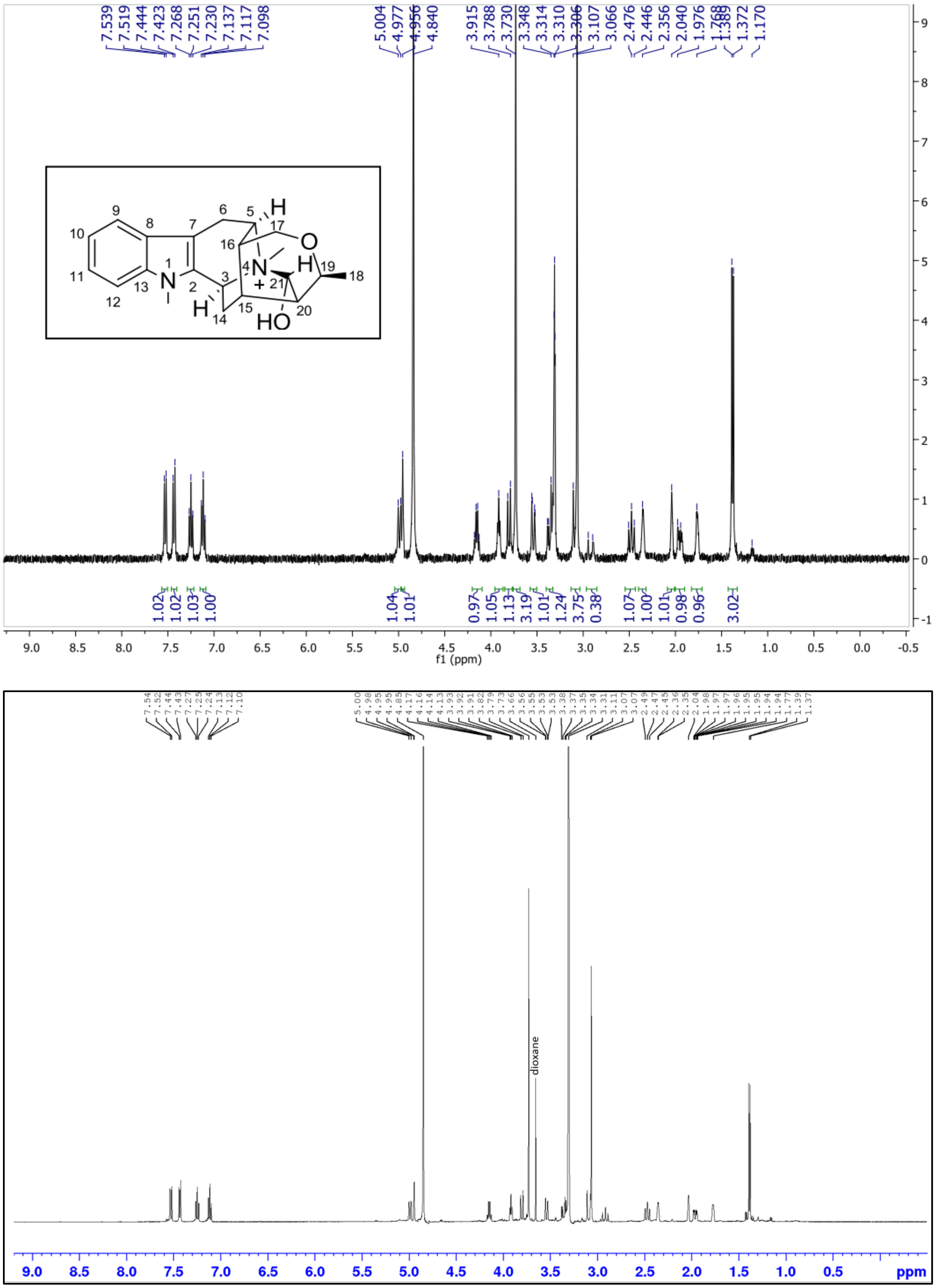

Figure S1. Comparison between the ^{1}H NMR spectra of natural[12] and synthetic N_4-methyl talpinine **12**.

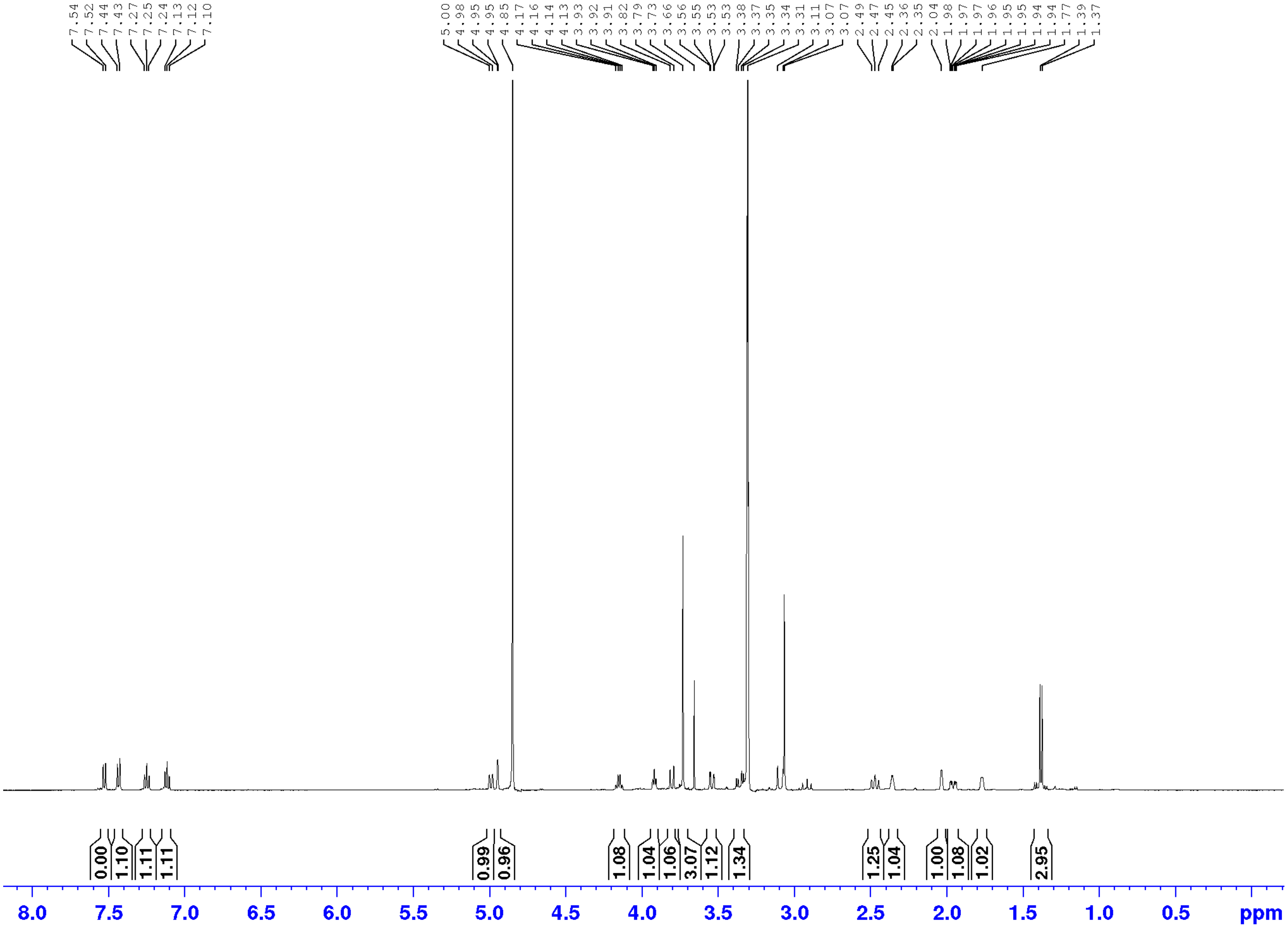

^{1}H NMR spectrum of synthetic *N*$_4$-methyltalpinine **12** (500 MHz, CD_3OD)

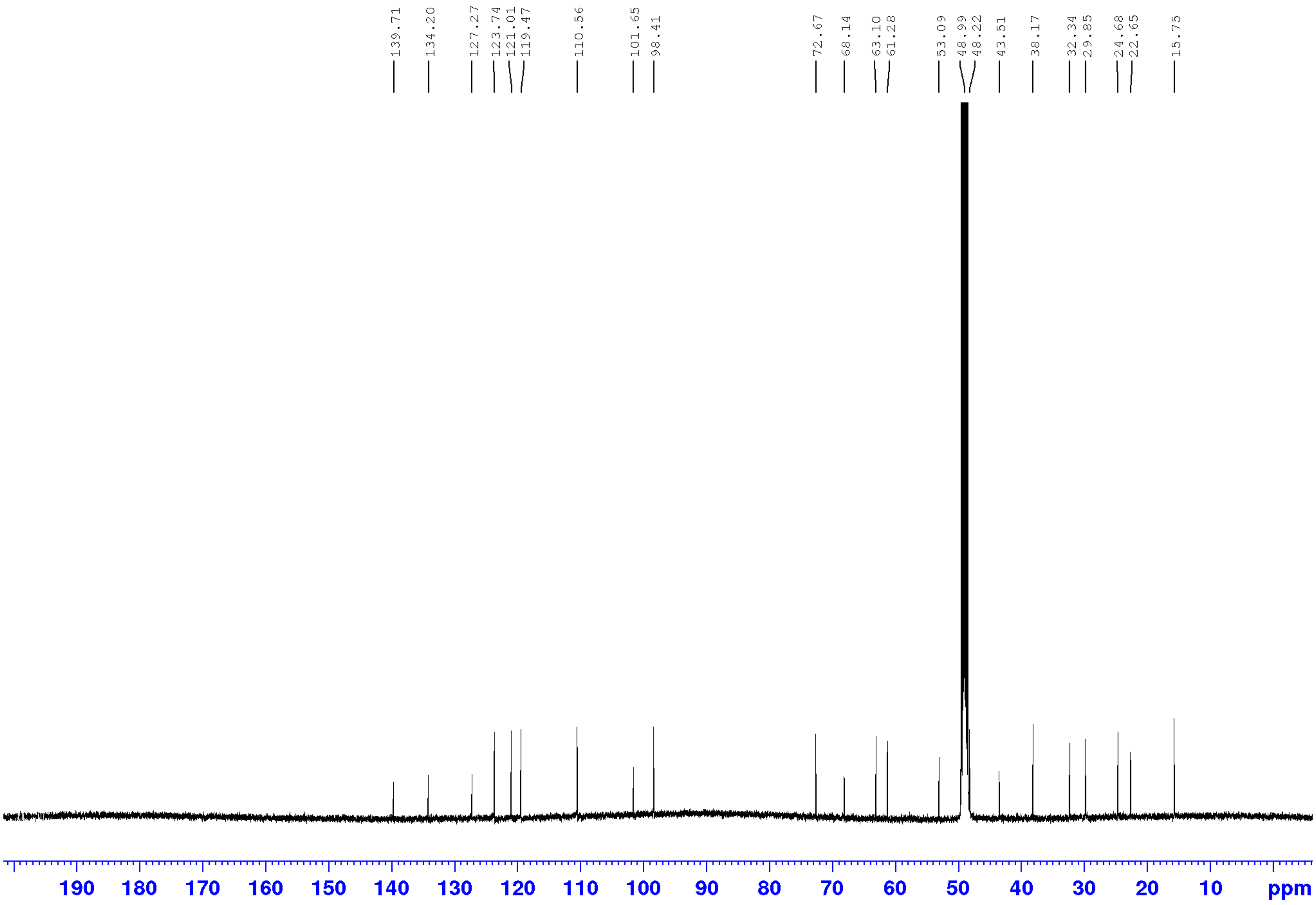

^{13}C NMR spectrum of synthetic *N*$_4$-methyltalpinine **12** (125 MHz, CD_3OD)

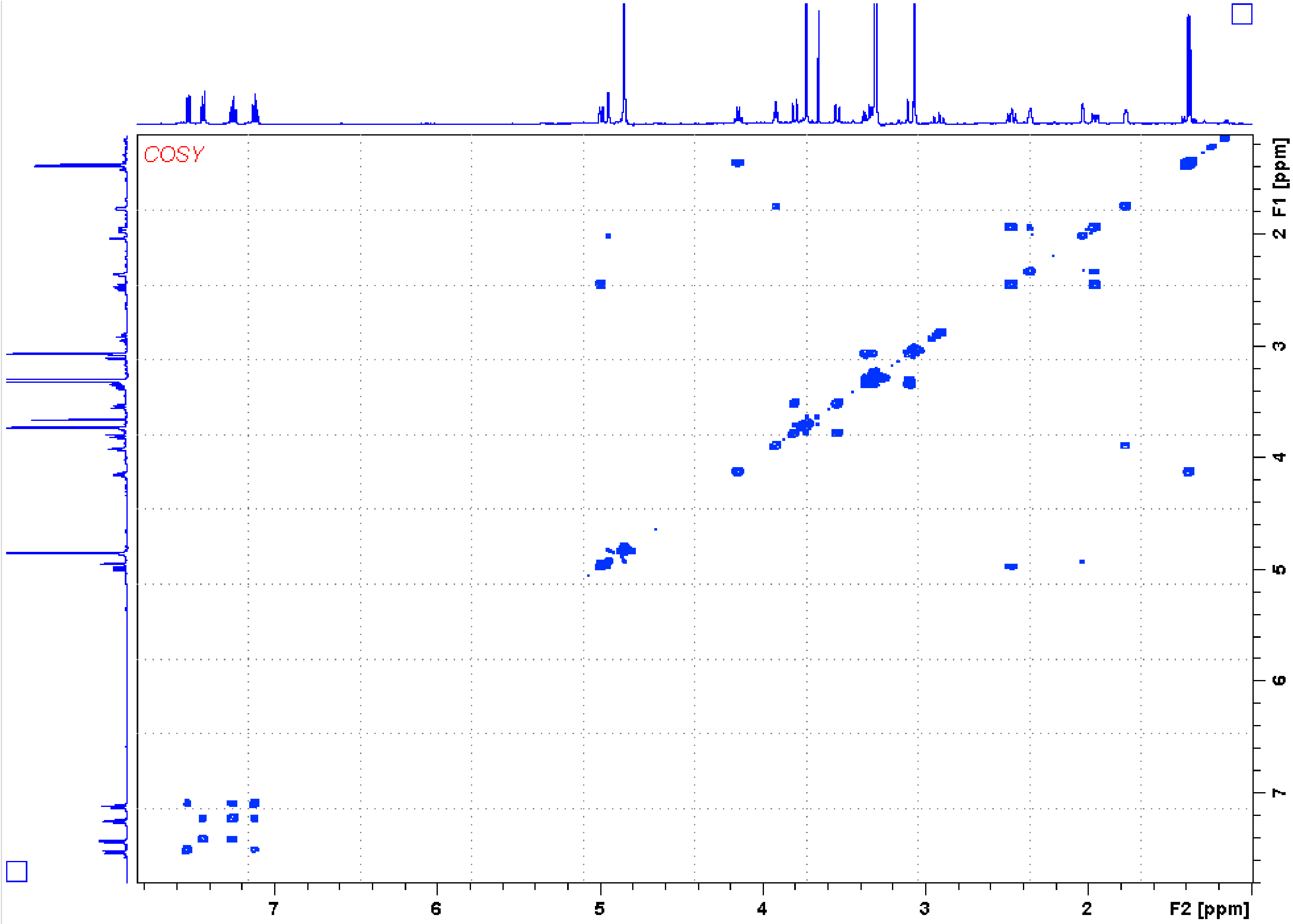

COSY NMR spectrum of synthetic N_4-methyltalpinine **12** (500 MHz, CD_3OD)

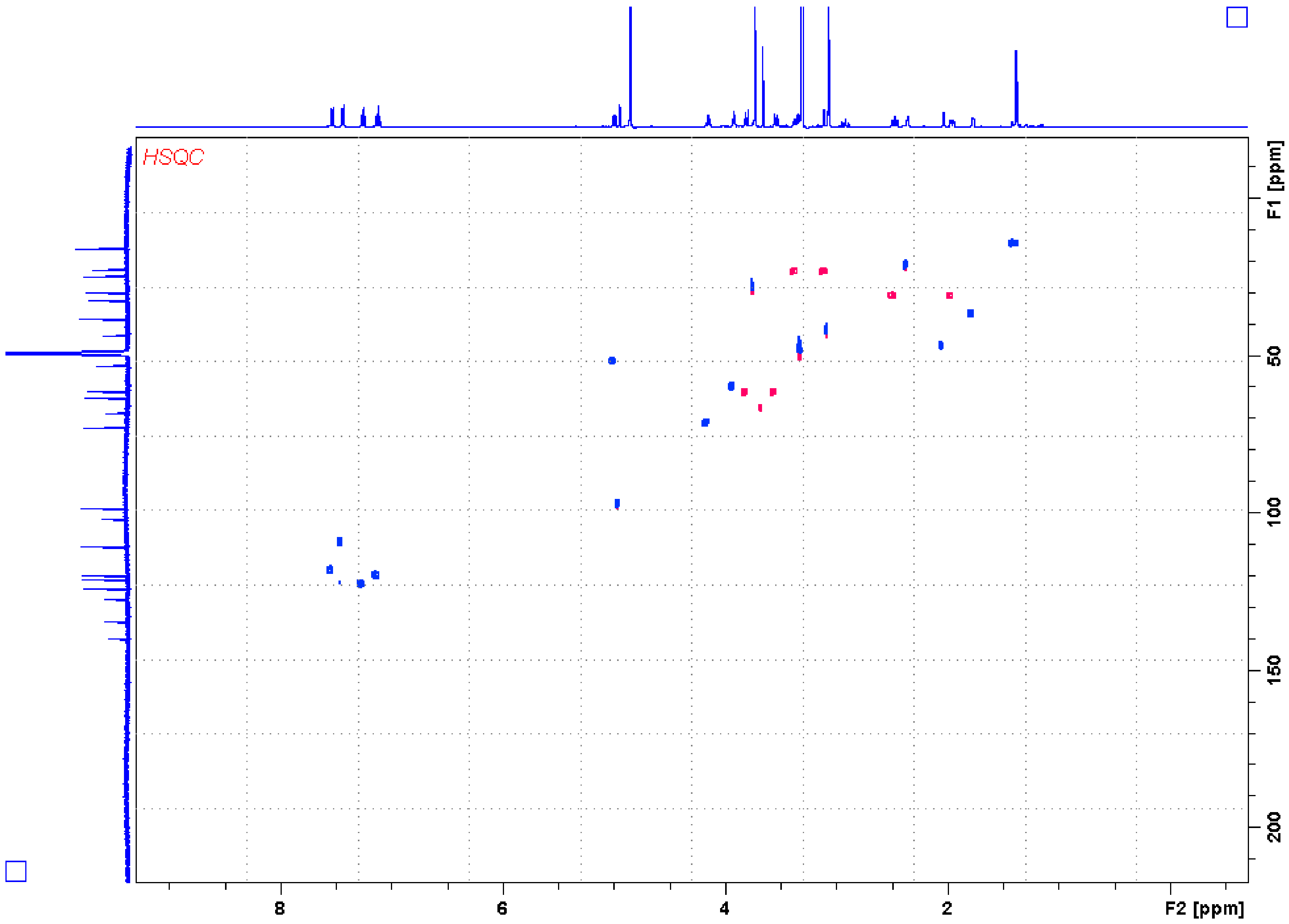

HSQC NMR spectrum of synthetic N_4-methyltalpinine **12** (500, 125 MHz, CD_3OD)

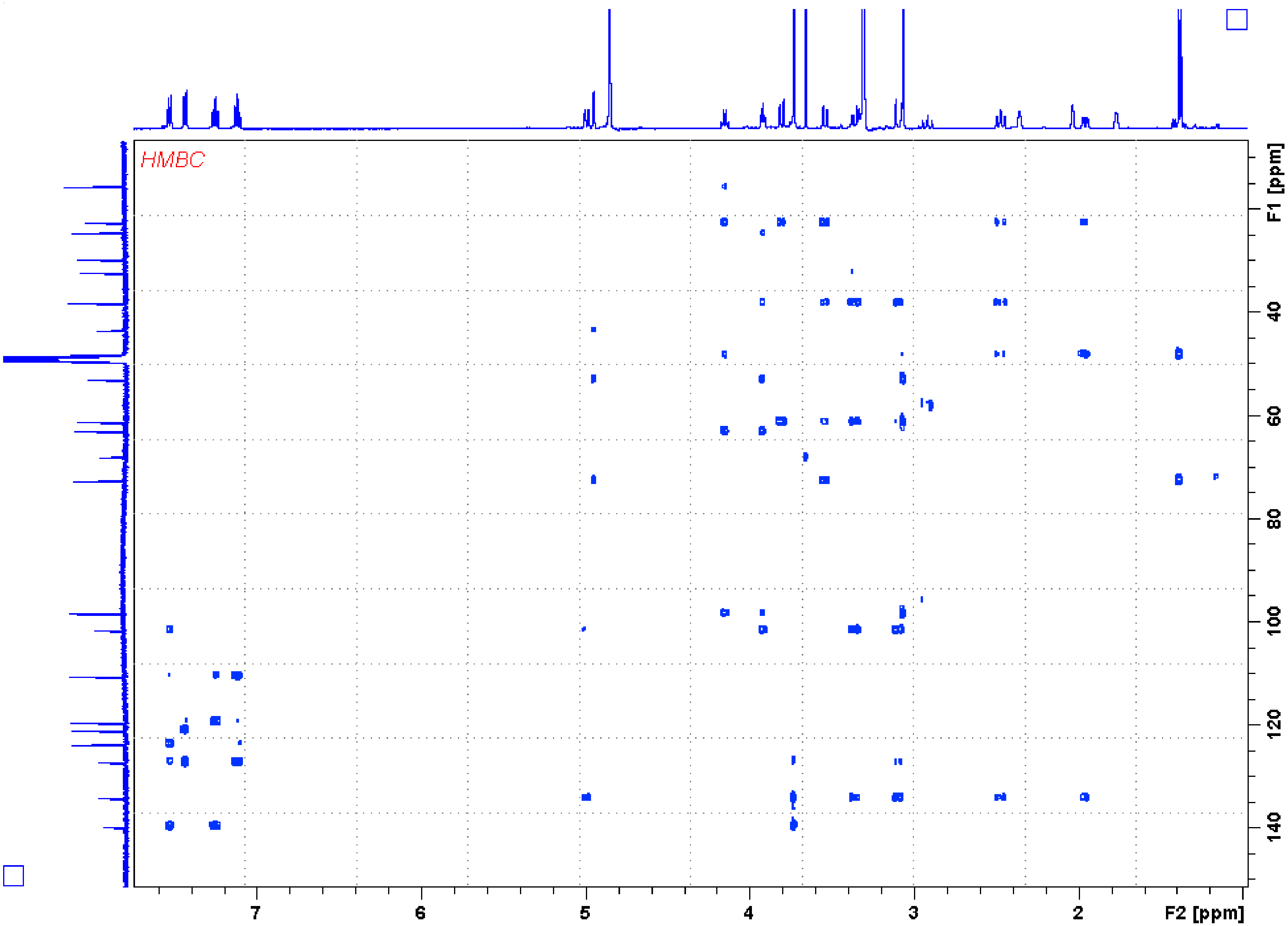

HMBC NMR spectrum of synthetic *N*4-methyltalpinine **12** (500, 125 MHz, CD_3OD)

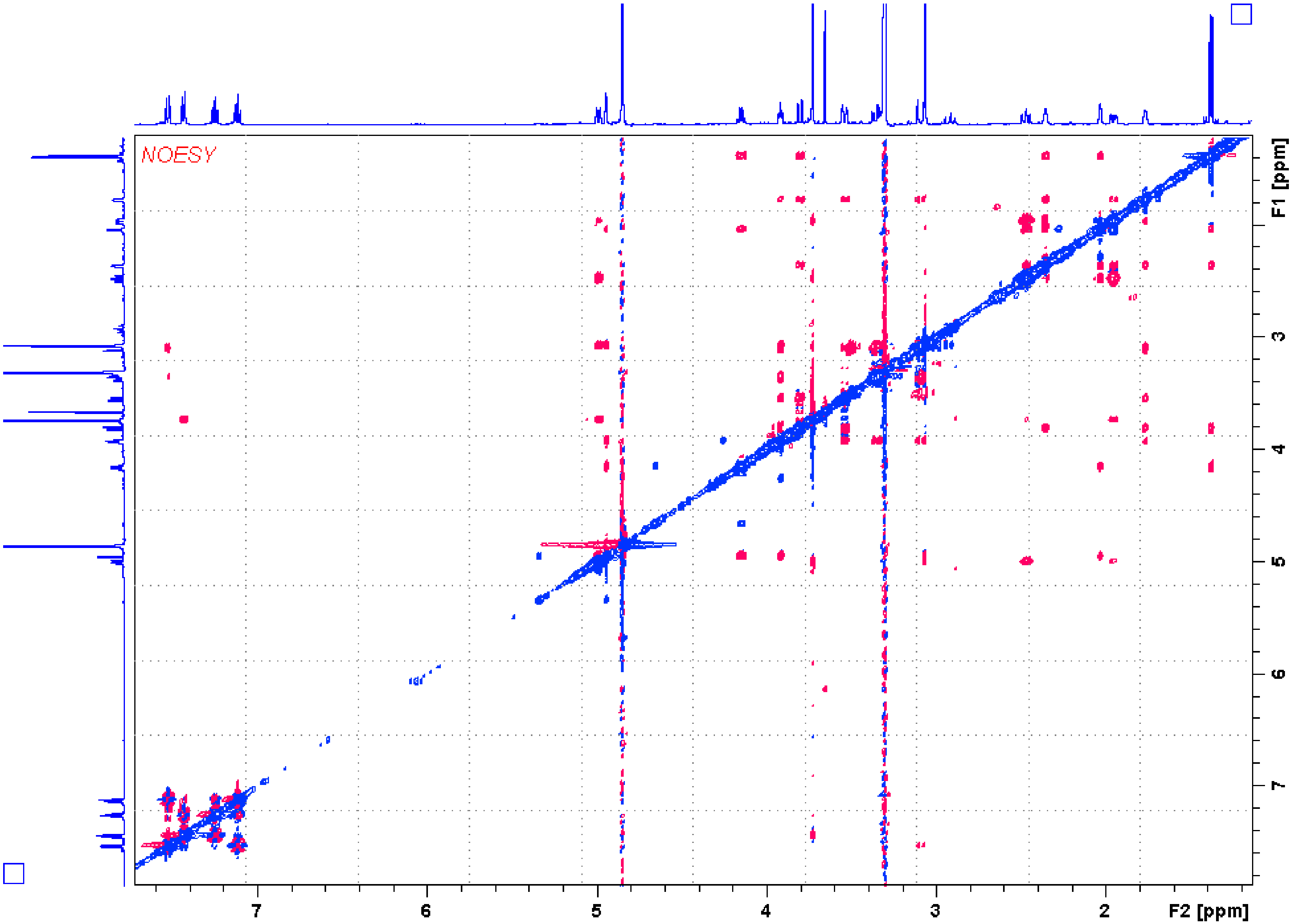

NOESY NMR spectrum of synthetic *N*4-methyltalpinine **12** (500 MHz, CD_3OD)
